# Time-restricted feeding reduces cardiovascular disease risk in obese mice

**DOI:** 10.1172/jci.insight.160257

**Published:** 2025-01-07

**Authors:** Paramita Pati, Carmen De Miguel, Jodi R. Paul, Dingguo Zhang, Jackson Colson, John Miller Allan, Claudia J. Edell, Megan K. Rhoads, Luke S. Dunaway, Sara N. Biswal, Yihan Zhong, Randee Sedaka, Telisha Millender-Swain, Shannon M. Bailey, Karen L. Gamble, David M. Pollock, Jennifer S. Pollock

**Affiliations:** 1 Section of Cardio-Renal Physiology & Medicine, Division of Nephrology, Department of Medicine;; 2 Division of Behavioral Neurobiology, Department of Psychiatry; and; 3 Division of Molecular and Cellular Pathology, Department of Pathology, Heersink School of Medicine, University of Alabama at Birmingham, Birmingham, Alabama, USA.

**Keywords:** Nephrology, Cardiovascular disease

## Abstract

Disrupted feeding and fasting cycles as well as chronic high-fat diet–induced (HFD-induced) obesity are associated with cardiovascular disease risk factors. We designed studies that determined whether 2 weeks of time-restricted feeding (TRF) intervention in mice fed a chronic HFD would reduce cardiovascular disease risk factors. Mice were fed a normal diet (ND; 10% fat) ad libitum or HFD (45% fat) for 18 weeks ad libitum to establish diet-induced obesity. ND or HFD mice were continued on ad libitum diet or subjected to TRF (limiting food availability to 12 hours only during the dark phase) during the final 2 weeks of the feeding protocol. TRF improved whole-body metabolic diurnal rhythms without a change in body weight. HFD mice showed reduced blood pressure dipping compared with ND, which was restored by TRF. Further, TRF reduced aortic wall thickness, decreased aortic stiffness, as well as increased kidney tubular brush border integrity, decreased renal medullary fibrosis, and reduced renal medullary T cell inflammation in HFD mice. These findings indicate that TRF may be an effective intervention for improving vascular and kidney health in a model of established diet-induced obesity.

## Abstract 2

Short-term time restricted feeding, initiated after establishing diet-induced obesity, improves cardiovascular and kidney health.

## Graphical abstract

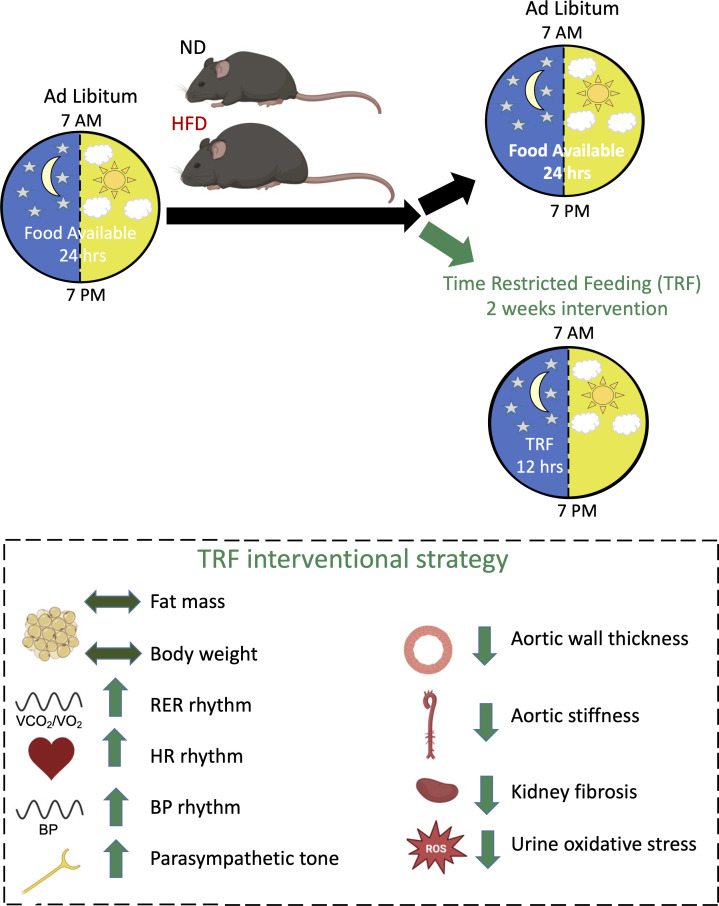

## Introduction

Feeding/fasting cycles influence the development of metabolic and vascular disease in humans (1) as well as physiological homeostasis (2, 3). Changes in a regular light/dark cycle and feeding/fasting cycles lead to neural and endocrine homeostatic dysfunction (4–7). For example, mice fed an ad libitum high-fat diet (HFD) have higher levels and altered rhythms of glucose and leptin, which are important metabolic regulators (7). Chronic ad libitum HFD mediates both aortic and kidney damage (6, 8–11) as well as contributing to the development of cardiometabolic disease (8–11).

Blood pressure (BP) and vascular function display diurnal rhythms (12–14) that are important for cardiovascular health. Loss of BP dipping is closely associated with target organ damage and cardiovascular disease (15). Both endothelial function and vascular resistance display light/dark variation in animal models (16, 17) and humans (13, 14), with endothelium-dependent relaxation as well as vascular resistance peaking during the active period (14, 16–18). HFD leads to endothelial dysfunction with loss of endothelial NO signaling in mice (19–21). Similarly, humans consuming an HFD have reduced endothelial function and increased cardiovascular disease risk (19, 22, 23). However, it is unknown whether feeding/fasting cycles with an HFD influence these cardiovascular disease risk factors.

Recent studies demonstrate the beneficial effects of time-restricted eating or feeding (TRF) on cardiometabolic health in humans and animals (24–35). In contrast with caloric restriction, TRF limits food intake to a specific time window but not the food content and thus induces increased fasting without reducing daily total caloric intake (36). Chronic TRF coincident with high-fat feeding reduces metabolic disease in rodents (30–35). In humans, TRF improves glucose levels, insulin sensitivity, cholesterol, oxidative stress, and BP (24–29) with food intake restricted to a daily time window without caloric restriction, suggesting that the timing of food intake and fasting is critical in the advancement and progression of disease.

We hypothesized that restricting food availability to the dark phase for a short intervention in a murine model of established diet-induced obesity would improve cardiovascular disease risk factors, specifically restoring BP dipping, decreasing kidney damage, as well as increasing aortic endothelial function and reducing aortic wall thickness and stiffness. To address this hypothesis, 8-week-old mice were fed ad libitum normal diet (ND) or HFD for 18 weeks followed by 2 weeks of ad libitum or TRF (food available only during the 12-hour dark phase) for a total of 20 weeks housed under similar conditions except for the food availability. During the final few days of the study, we measured metabolic rhythms, hemodynamics, diurnal aortic vascular reactivity, aortic and kidney pathology, and circulating mediators in all 4 diet and feeding groups. The results indicate that 2 weeks of TRF in a mouse model of established HFD-induced obesity is sufficient to reduce cardiovascular disease risk and improve vascular health compared with ad libitum HFD independent of body weight.

## Results

### TRF reinstates whole-body metabolic rhythms independent of weight gain in HFD-fed mice.

**Figure 1:**
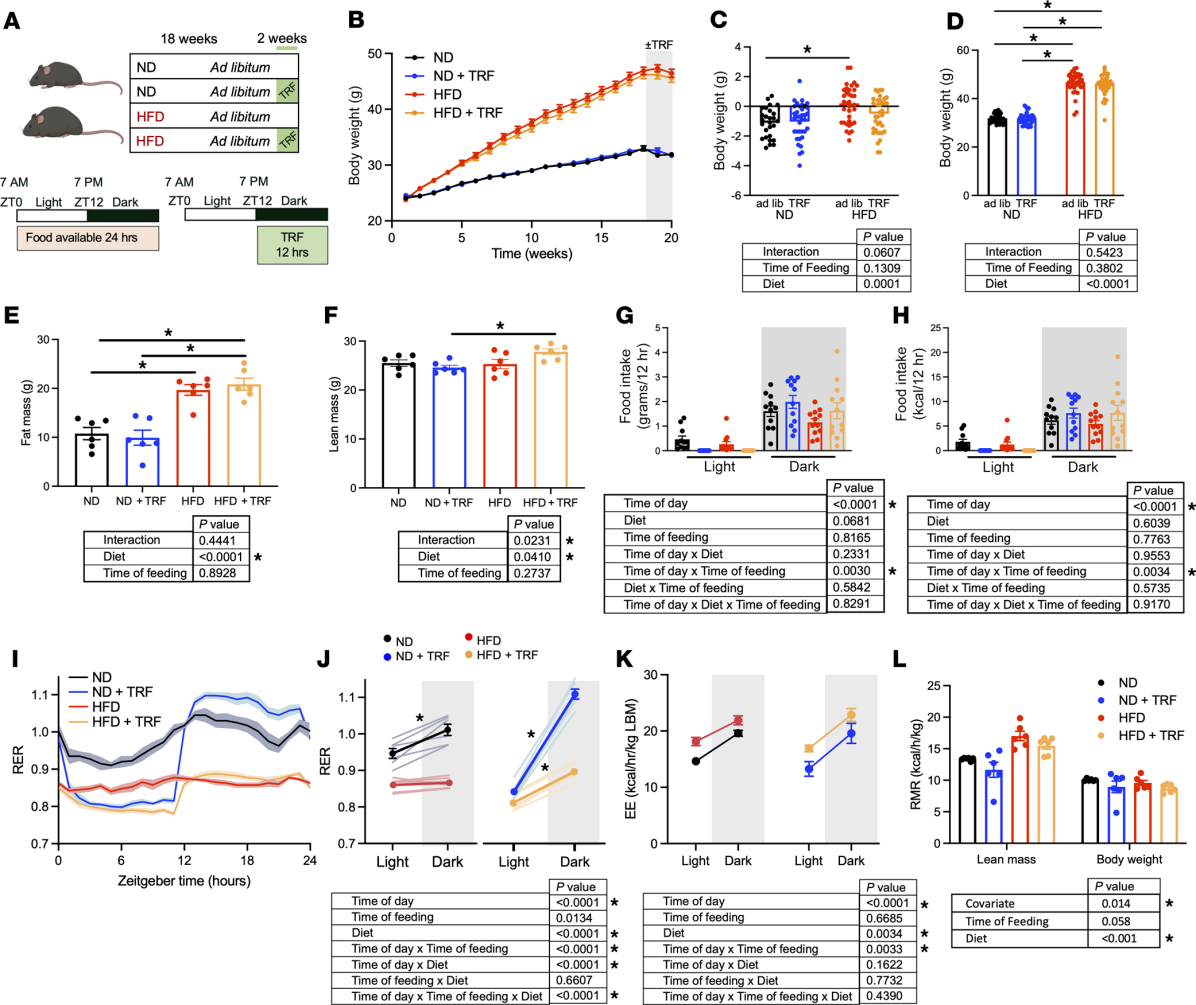
TRF institutes whole-body metabolic rhythms independent of weight gain. (**A**) Experimental design of the dietary protocol depicts 18 weeks of ad libitum feeding followed by 2 weeks of ad libitum food availability or TRF. ZT, Zeitgeber time. (**B**) Longitudinal body weight gain from week 1 to week 18 of ND and HFD followed by week 19 and week 20 of ND (*n* = 35), ND+TRF (*n* = 35), HFD (*n* = 36), and HFD+TRF (*n* = 36) mice maintained in home cages. (**C**) Change in body weight from week 18 to week 20 from mice maintained in home cages (*P < 0.05, 2-way ANOVA). (**D**) Body weights at week 20 in mice maintained in home cages (*P < 0.05, 2-way ANOVA was used to compare diet and time of feeding). Quantitative magnetic resonance (QMR) measurements of (**E**) fat mass and (**F**) lean mass (2-way ANOVA was used to compare diet and time of feeding, *n* = 6, *P < 0.05). Food intake was measured during light and dark phases in metabolic cages. Food intake in g/12 h (**G**) and in kcal/12 h (**H**) measured at ZT0 and ZT12. Three-way repeated measures ANOVA was used to compare diet, time of feeding, and time of day (*n* = 11–12, *P < 0.05). Whole-body metabolic rhythms were measured by Comprehensive Lab Animal Monitoring System (CLAMS) in a separate cohort of mice. Data are shown as the 24-hour average profile of (**I**) respiratory exchange ratio (RER) and (**J**) RER light and dark phase differences. (**K**) Energy expenditure (EE) was determined relative to lean body mass in light and dark phases. Three-way repeated measures ANOVA was used to compare diet, time of feeding, and time of day (*n* = 6, *P < 0.05). (**L**) Resting metabolic rate (RMR) was calculated. Two-way ANCOVA with lean body mass as the covariate was used (*n* = 6, *P < 0.05).

Mice fed an ND or HFD were subjected to 18 weeks of ad libitum feeding, followed by 2 weeks with food available ad libitum in the control groups or only during the 12-hour dark phase in the TRF groups (Figure 1A) for a total of 20 weeks of feeding. Sham control mice were housed alongside the TRF mice and were exposed to the same disturbance by lab personnel at specific times of day, including having their cage and food container similarly handled as the TRF mice, except that food remained in the container, thus controlling for any confounders related to the TRF protocol. Mice on HFD had significantly higher body weights than mice on ND (Figure 1, B and D). Most mice in this study were group-housed in home cages, with subsets of mice singly housed for specific measurements. Interestingly, body weight was not affected by the 2-week TRF period in both diet groups of mice in group-housed home cages or in singly housed specialty cages (Figure 1, B–D and Supplemental Figure 1; supplemental material available online with this article; https://doi.org/10.1172/jci.insight.160257DS1). Quantitative magnetic resonance (QMR) measurements revealed significantly higher fat mass in mice on HFD compared with ND groups regardless of TRF (Figure 1E). Lean mass was also significantly higher in HFD+TRF mice (Figure 1F). Measurements of food consumption during the 12-hour light and dark phases were monitored during the final 3 days of the dietary and feeding protocol after an acclimation period with mice in the singly housed metabolic cages. There was an obvious difference in the food consumption during the light period in the TRF groups of both diets compared with the ad libitum groups (Figure 1, G and H), since there was no food available in the light period for the TRF groups. Food consumption during the dark phase did not significantly differ among the 4 dietary and feeding groups of mice, though there was a trend for the HFD groups to consume less food (Figure 1, G and H). Daily (24-hour) food and caloric intake was monitored in both the singly housed metabolic cages as well as group-housed home cages. TRF did not influence the daily food intake or the daily caloric intake in either diet group in the singly housed cages (Supplemental Figure 2). Mice on HFD in home cages showed significantly decreased daily food intake but not caloric intake compared to the ND mice in home cages (Supplemental Figure 2). TRF did not influence daily food intake or caloric intake in ND mice in home cages (Supplemental Figure 2). TRF significantly increased daily food intake and caloric intake in HFD mice in home cages (Supplemental Figure 2).

We examined the whole-body respiratory exchange ratio (RER) profile continuously over 24 hours to determine whether TRF would improve this metabolic rhythm (Figure 1I). RER is the ratio of the volume of CO_2_ produced and the volume of O_2_ consumed and as such is not normalized to body weight or composition. ND mice displayed a diurnal variation in RER (*P* = 0.004; Figure 1, I and J) as expected. HFD mice showed no difference in RER between light and dark phases (Figure 1, I and J). Surprisingly, TRF significantly increased the RER light/dark difference in both ND and HFD mice (ND, *P* < 0.001; HFD, *P* < 0.001; Figure 1, I and J). Energy expenditure (EE) normalized to lean body mass was significantly higher in HFD compared with ND mice (main effect of diet, *P* = 0.005; Figure 1K), and TRF increased the EE light/dark difference in both ND and HFD mice (time of day × time of feeding interaction, *P* = 0.002; Figure 1K). Resting metabolic rate (RMR) normalized to lean body mass was significantly higher in HFD mice compared with ND mice (main effect of diet, *P* < 0.001) with a trend toward decreased RMR with TRF in ND and HFD mice (main effect of time of feeding, *P* = 0.058; Figure 1L).

### TRF improves BP and heart rate dipping and rhythms.

**Figure 2:**
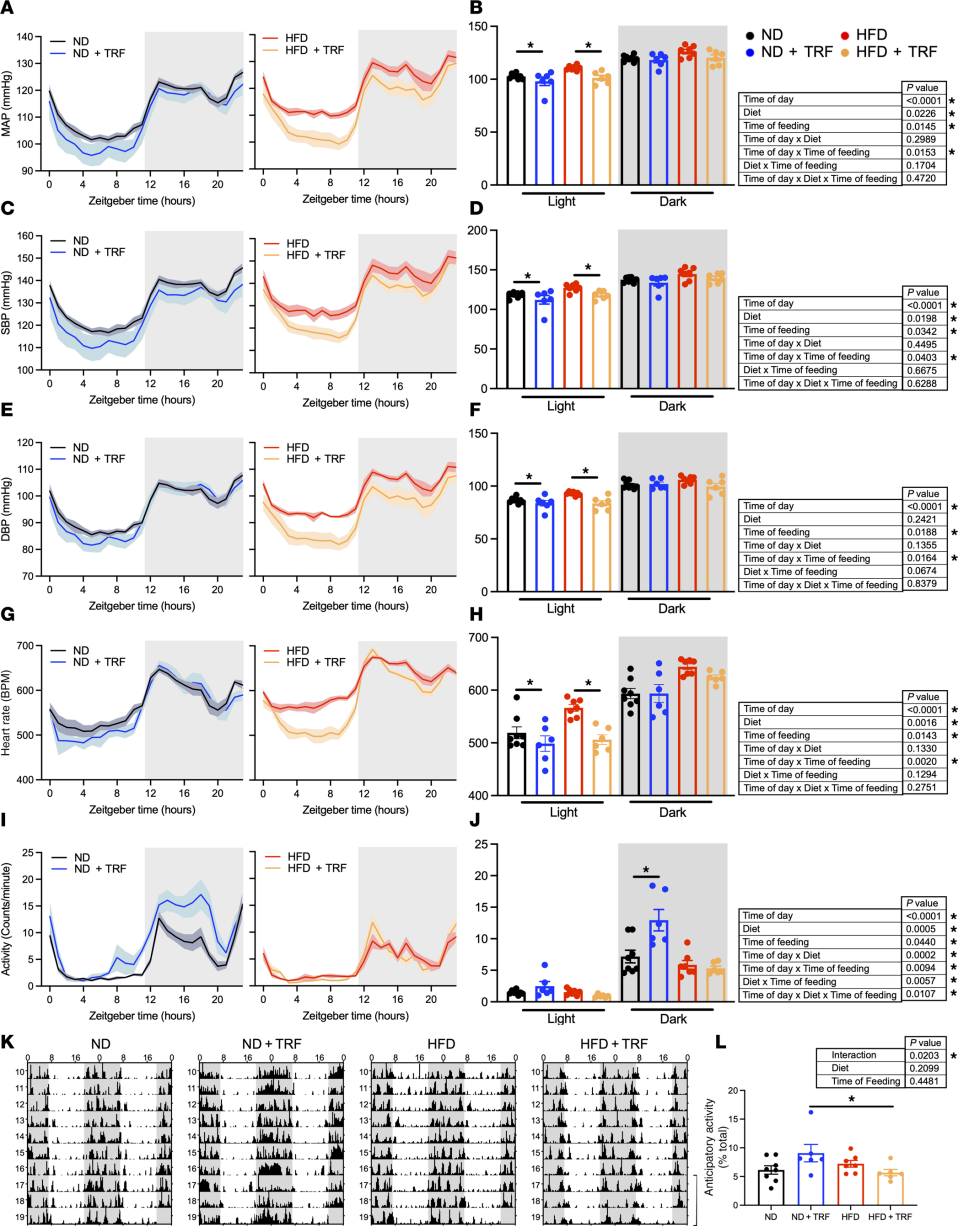
TRF improves light-phase BP and heart rate changes. Radiotelemetry was used to measure BP, heart rate (HR), and locomotor activity continuously in unrestrained, conscious mice. Light and dark phases are indicated by white and gray shading, respectively. Telemetry traces of the last 3 days’ average of (**A**) mean arterial pressure (MAP), (**C**) systolic blood pressure (SBP), (**E**) diastolic blood pressure (DBP), (**G**) HR, and (**I**) locomotor activity are shown. Individual mouse BP, HR, and activity for the light and dark phase were assessed by calculating the average from ZT3–ZT10 and ZT15–ZT22 periods, as well as the difference between these periods. Eight-hour averages of light and dark phases with main effects and interactions are shown in **B**, **D**, **F**, **H**, and **J**. Two-way ANOVA was used to compare diet and time of feeding during the light or dark period (*P < 0.05 ad lib vs. TRF). Three-way repeated measures ANOVA was used to compare diet, time of feeding, and time of day (*n* = 6–8, *P < 0.05). (**K**) Representative actograms of locomotor activity generated with ClockLab (Actimetrics) are shown. Bracket indicates the 3 days used for analysis in **A**–**J** and **L**. (**L**) Food anticipatory activity was assessed from telemetry activity data using activity occurring from ZT8–ZT12 (as percentage of total daily activity). Two-way ANOVA was used to compare diet and time of feeding (*n* = 6–8, *P < 0.05).

We sought to examine whether TRF would improve BP in HFD mice. BP follows a well-established diurnal rhythm of lower BP during the light phase compared with the dark phase in mice (Figure 2 and Supplemental Table 1). We found that the chronic HFD blunted mean arterial pressure (MAP), systolic blood pressure (SBP), and diastolic blood pressure (DBP) dipping compared with ND mice (Figure 2, A–F). TRF reinstated MAP, SBP, and DBP dipping in ND and HFD mice (Figure 2, A–F; ΔMAP: ND, 17 ± 1.0 mmHg; ND+TRF, 20 ± 2.1 mmHg; HFD, 15 ± 1.2 mmHg; HFD+TRF, 19 ± 0.9 mmHg; *n* = 6–8). Cosinor analysis of BP rhythms verified these results, showing that TRF increases the amplitude of MAP, SBP, and DBP without changes in mesor or acrophase in the HFD mice (Supplemental Table 1).

Diurnal variation in HR was observed in all groups. However, HFD mice showed increased HR during both light and dark phases (Figure 2, G and H). TRF significantly reduced HR in ND and HFD mice during the light phase (*P* = 0.008; Figure 2, G and H). TRF also increased the light/dark HR difference (ΔHR: ND, 70 ± 4.7 bpm; ND+TRF, 94 ± 9.1 bpm; HFD, 74 ± 9.5 bpm; HFD+TRF, 119 ± 10.3 bpm; *n* = 6–8; 2-way ANOVA results: Time of feeding, *P* < 0.01; HFD vs. HFD+TRF, *P* = 0.007).

Time domain analysis of HR variability revealed that time of day, diet, and time of feeding altered beat-to-beat interval (N-N interval) (Supplemental Table 2). The standard deviation of the N-N interval (SDNN) was lower in HFD mice compared with ND mice, with TRF improving the light/dark difference in SDNN in a time of day–dependent manner independent of dietary fat content. The root mean of SDNN (reflecting vagal mediation of HR variability) was also lower in HFD than ND mice and was improved during the light phase by TRF, though not restored to the level observed in ND mice. The percentage of N-N intervals exceeding 6 ms was also lower in HFD mice.

Locomotor activity measured by telemetry displayed a diurnal rhythm in all 4 groups of mice, being highest in the dark phase as expected (Figure 2I). Mice eating an HFD displayed lower locomotor activity than mice on an ND. TRF significantly increased activity in ND mice with no change in HFD mice (Figure 2, I and J). The light/dark difference in activity was significantly increased in ND+TRF mice (ΔActivity: ND, 5.7 ± 1.0 counts/min; ND+TRF, 10.2 ± 1.1 counts/min; HFD, 4.3 ± 0.6 counts/min; HFD+TRF, 4.4 ± 0.3 counts/min; *n* = 6–8; 2-way ANOVA results: Diet, *P* < 0.01; Time of feeding, *P* = 0.01; Interaction, *P* = 0.02; ND vs. ND+TRF, *P* = 0.006). Patterns of locomotor activity appeared to be similar among ND, HFD, and HFD+TRF mice, as shown in representative actograms (Figure 2K). Interestingly, TRF significantly increased food anticipatory activity in ND mice compared with HFD mice (Figure 2L).

### TRF restores aortic function and reduces aortic wall thickness in HFD-fed mice.

**Figure 3:**
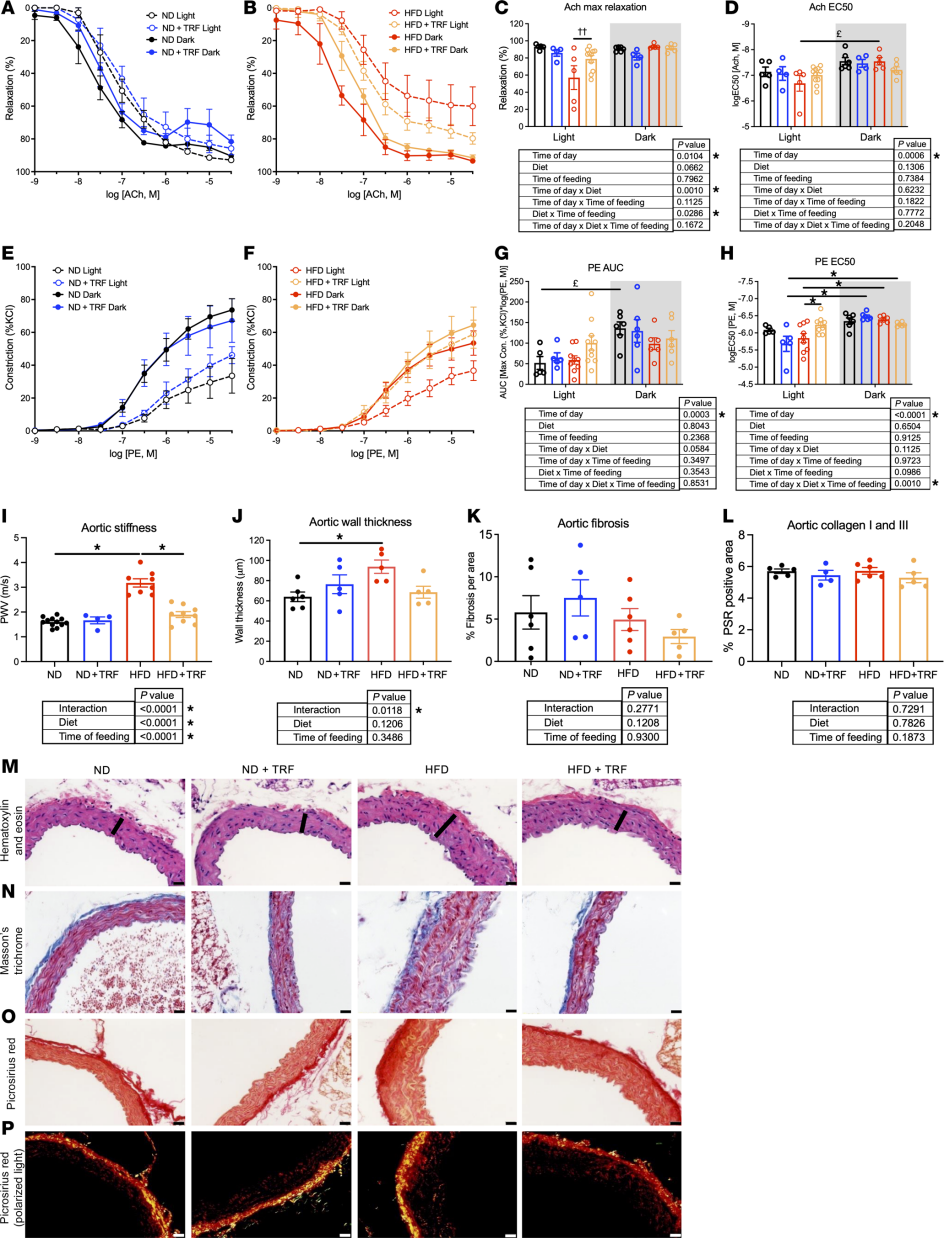
TRF restores aortic endothelial function and reduces aortic damage in HFD mice. Vascular function studies in thoracic aortas were conducted with cumulative concentration–response curves of Ach, SNP, PE, and KCl. Ach-induced vascular relaxation during the light and dark phases is shown (**A** and **B**), along with (**C**) E_max_ and (**D**) EC_50._ PE-induced vasoconstriction curves in the light and dark phases are shown (**E** and **F**), along with (**G**) AUC and (**H**) EC_50_. Two-way ANOVA was used to compare diet and time of feeding during the light or dark period (^††^*P* < 0.05 HFD and HFD+TRF light vs. HFD and HFD+TRF dark). Three-way ANOVA was used to compare diet, time of feeding, and time of day with Holm-Šídák post hoc test (*n* = 5–10, *P < 0.05; ^£^*P* < 0.05 light vs. dark). Main effects and interactions are shown below graphs. (**I**) Aortic stiffness was assessed with PWV measurements between ZT2 and ZT5 in a separate cohort of mice. Two-way ANOVA was used to compare diet and time of feeding (*n* = 4–12, *P < 0.05). (**M**) A different cohort of mice sacrificed at ZT17 was used for histological analysis. Aortic vascular remodeling is shown with representative images, scale bar = 20 μm. Representative aortic cross sections were stained with H&E, and aortic wall thickness, an index of medial hypertrophy, was measured (**J**). Changes in wall thickness are indicated with a black line. (**N**) Representative aortic sections were stained with Masson’s trichrome, and (**K**) fibrosis was quantified in the 4 groups of mice. (**O**) Representative aortic cross sections were stained with Picrosirius red. (**P**) Picrosirius red–stained sections were examined under polarized light for (**L**) quantification of collagen I and III content. Two-way ANOVA was used to compare diet and time of feeding (*n* = 4–6, *P < 0.05).

Endothelium-dependent and -independent vasorelaxation were assessed in thoracic aorta isolated during both light and dark phases (ZT0 and ZT12) based on responses to acetylcholine (Ach) and sodium nitroprusside (SNP), respectively (Figure 3, A and B, and Table 1). Endothelium-independent relaxation, assessed by the NO donor SNP, was similar in all groups during both the light and dark phases (Table 1), indicating that the endothelium was specifically impaired by this model of chronic HFD. Endothelial function was similar in ND and ND+TRF mice. In HFD mice, Ach-mediated vasorelaxation was significantly reduced in the light compared with the dark phase (*P* < 0.001; Figure 3C). In general, Ach-induced maximum vasorelaxation (E_max_) was significantly impaired in ad libitum–fed HFD mice compared with ND mice (*P* = 0.006) and tended to be improved by TRF (*P* = 0.061; Figure 3C). Sensitivity to Ach (logEC_50_) showed a light/dark difference, though no effects of diet or time of feeding were observed (Figure 3D).

We also determined aortic vasoconstriction responses to PE and KCl during the light and dark phases (Figure 3, E and F, and Table 1). In ND mice, PE-induced vasoconstriction was greater during the dark compared with the light phase (Figure 3G). The diurnal difference of PE-induced vasoconstriction was maintained in all groups of mice, as time of day had a significant main effect (Figure 3G); however, TRF of HFD mice increased sensitivity to PE-induced vasoconstriction during the light phase (Figure 3H). KCl-induced vasoconstriction was not different among groups (Table 1).

Aortic pulse wave velocity (PWV), a determinant of aortic function, was also assessed in the 4 groups of mice. PWV is a measure of aortic stiffness. Aortic stiffness, an indicator of cardiovascular disease, is known to be highly associated with obesity. We found that aortic PWV was significantly higher in HFD mice than in ND mice (Figure 3I). TRF restored the PWV in HFD mice to that observed in ND mice. Aortic PWV was similar between ND and ND+TRF mice (Figure 3I). We also determined whether TRF would impact aortic wall thickness and fibrosis in HFD mice (Figure 3, J, K, M, and N). Aortic wall thickness was significantly increased in HFD mice compared with ND mice. A significant interaction between diet and time of feeding was also observed, largely driven by lower wall thickness in the HFD+TRF group (Figure 3J). Neither dietary fat nor time of feeding altered the extent of aortic fibrosis apparent among the different groups of mice (Figure 3K). Similarly, we did not find changes in type I and III collagen content assessed by Picrosirius red staining among groups (Figure 3, L, O, and P).

### TRF improves kidney pathology in HFD mice.

**Figure 4:**
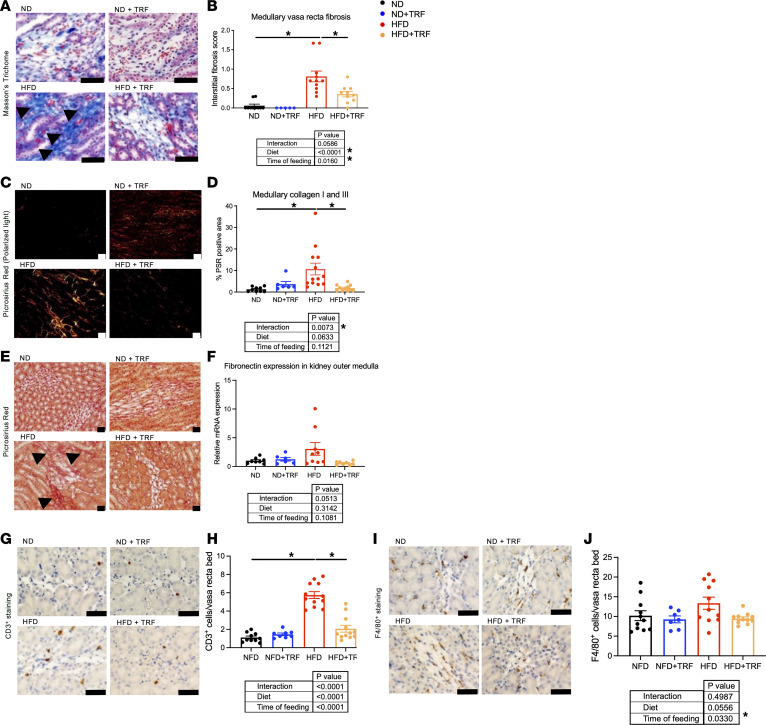
TRF reduces measures of kidney damage to the kidney medullary vasa recta area in HFD mice. Representative histological images, quantitative histological scoring of kidney damage, medullary fibronectin gene expression, and representative immunohistochemical images and quantitative analyses of kidney immune cells in ND, ND+TRF, HFD, and HFD+TRF mice. Representative images and scoring of kidney medullary vasa recta region sections. (**A**) Masson’s trichrome staining, (**B**) fibrosis histological scores, (**C**) Picrosirius red staining under polarized light, (**D**) quantitation of collagen I and III content, (**E**) Picrosirius red staining under brightfield light. Arrows indicate vasa recta fibrosis in **A** and **E**. (**F**) Kidney medullary fibronectin mRNA expression. Representative images of medullary vasa recta region with immunohistochemical staining for (**G**) CD3^+^ cells and (**I**) immunohistochemical staining for F4/80^+^ cells. Scale bar = 50 μm. Brown staining indicates CD3^+^ cells and F4/80^+^ cells. Quantitative analysis of (**H**) CD3^+^ cells per medullary vasa recta bed and (**J**) F4/80^+^ cells per medullary vasa recta bed. Scale bar = 50 μm. Two-way ANOVA was used to compare diet and time of feeding with Tukey’s post hoc test for histological scoring assessments (*n* = 5–13, *P < 0.05).

**Figure 5:**
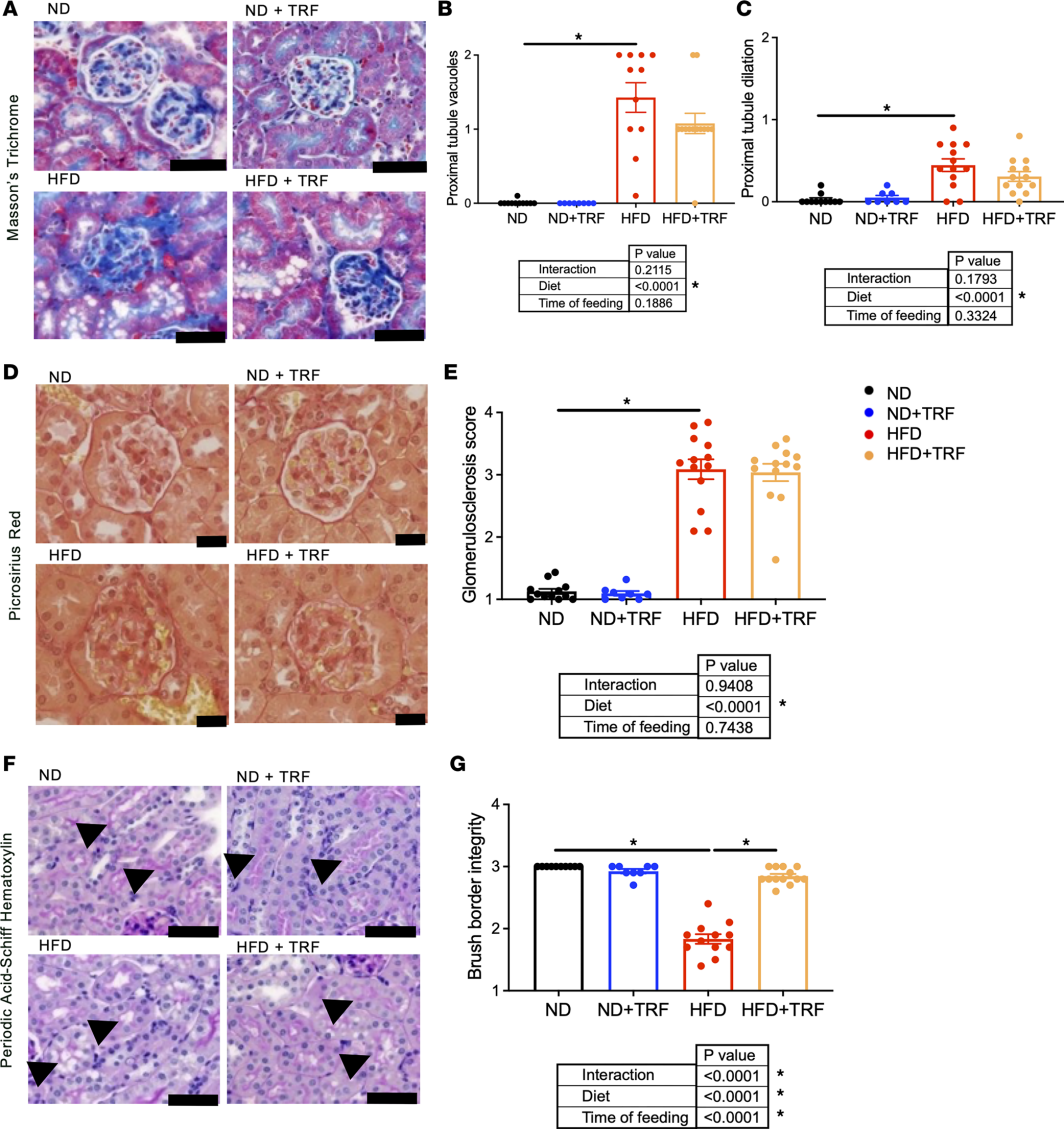
TRF reduces measures of kidney damage to glomeruli and cortical tubules in HFD mice. Representative images of kidney cortex sections. (**A**) Masson’s trichrome staining, (**B**) proximal tubule vacuole scores, (**C**) proximal tubule dilation scores, (**D**) Picrosirius red staining under brightfield light, (**E**) glomerulosclerosis histological scores, (**F**) Periodic acid–Schiff hematoxylin-staining, (**G**) proximal tubule brush border integrity scores. Arrows indicate proximal tubule brush border in **F**. Scale bar = 50 μm. Two-way ANOVA was used to compare diet and time of feeding with Tukey’s post hoc test for histological scoring assessments (*n* = 5–13, *P < 0.05).

Reduced BP dipping is an indicator of target organ damage. Kidney damage is also a consequence of diet-induced obesity; thus, we sought to determine the effect of TRF on kidney pathology. Chronic ad libitum HFD induced fibrotic deposition in the vasa recta vascular beds of the kidney medulla (Figure 4, A and B). In addition, TRF reduced type I and III collagen deposition in the medulla of HFD mice (Figure 4, C–E) and reduced outer medullary fibronectin mRNA expression (interaction, *P* = 0.0513; Figure 4F) in HFD mice. Histological assessments verified that, compared with ND mice, chronic HFD mice exhibited renal cortical damage evidenced by the appearance of vacuoles in proximal tubular cells, proximal tubule dilation, and the presence of glomerulosclerosis (Figure 5, A–E) as well as decreased proximal tubular brush border integrity (Figure 5, F and G). TRF normalized proximal tubular brush border integrity (Figure 5, F and G) and tended to reduce the abundance of proximal tubule vacuoles (Figure 5, A and B). However, TRF did not improve glomerulosclerosis in HFD mice (Figure 5, D and E).

Assessment of renal immune cell infiltration revealed that HFD mice had significantly elevated numbers of CD3^+^ cells (T lymphocytes) within the medullary vasa recta region compared with ND mice. TRF significantly reduced T lymphocytes in HFD mice (Figure 4, G and H). However, F4/80^+^ (macrophage) cell numbers in this same region did not differ significantly among the 4 groups of mice, although the TRF groups were generally lower than ad libitum–fed mice (Figure 4, I and J).

**Figure 6:**
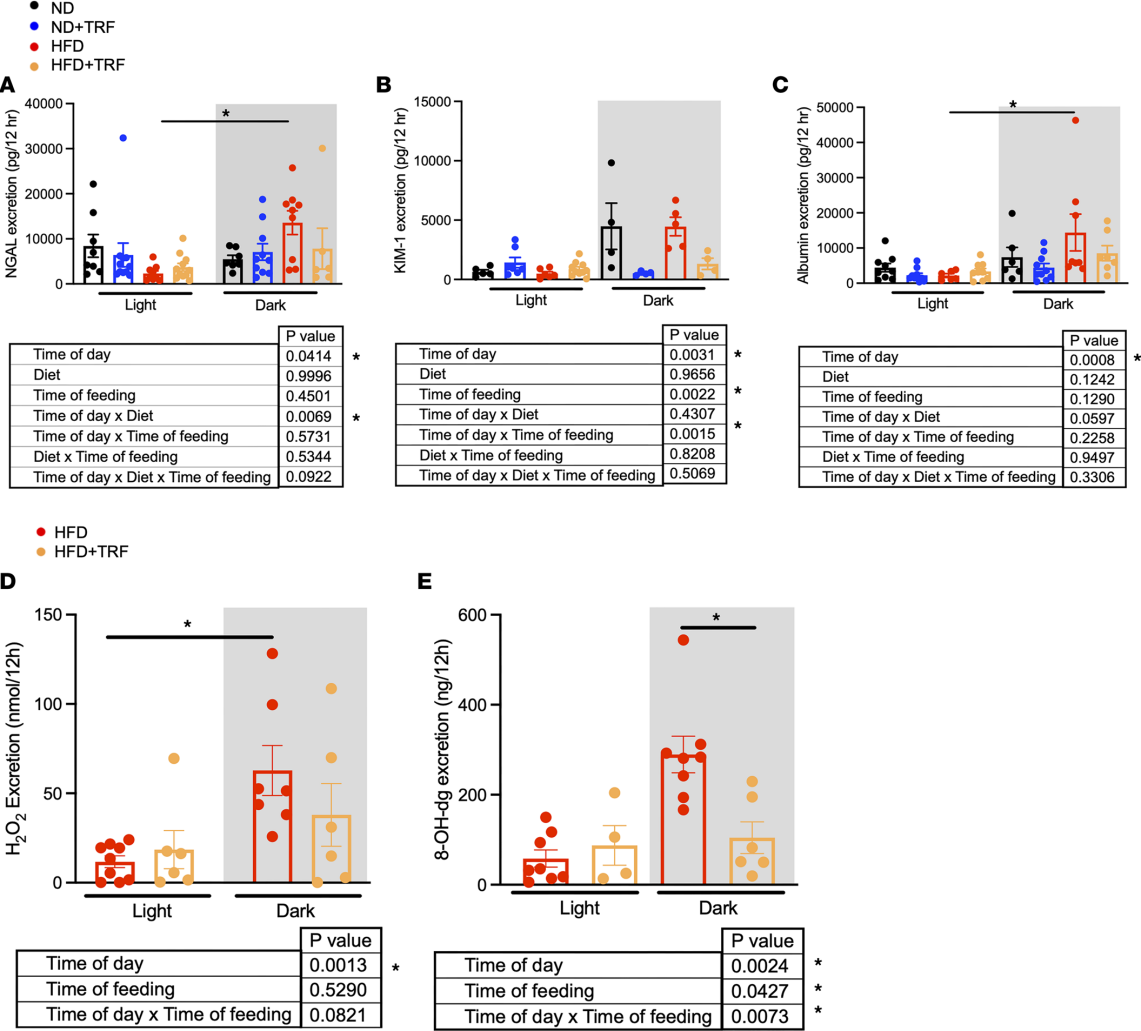
TRF reduces the urinary excretion of kidney damage markers in HFD mice. Light- and dark-phase (12-hour) urinary excretion of kidney damage markers (**A**) NGAL, (**B**) KIM-1, and (**C**) albumin from ND, ND+TRF, HFD, and HFD+TRF mice. Three-way repeated measures ANOVA was used to compare diet, time of feeding, and time of day (*n* = 5–12, *P < 0.05). Light- and dark-phase (12-hour) urinary excretion of oxidative stress markers (**D**) H_2_O_2_ and (**E**) 8-OHdG from HFD and HFD+TRF mice. Two-way ANOVA was used to compare time of day and time of feeding with Holm-Šídák post hoc test (*n* = 4–9, *P < 0.05).

To further assess HFD-induced kidney injury, urinary excretion of the damage markers neutrophil gelatinase-associated lipocalin (NGAL), kidney injury marker 1 (KIM-1), and albumin were measured during the light and dark phases. NGAL excretion showed a day/night difference that was higher during the dark phase in ad libitum–fed HFD mice (Figure 6A). TRF evoked similar reductions in dark-phase KIM-1 excretion by both diet groups (Figure 6B). Urinary albumin excretion was significantly higher during the dark phase but did not differ among groups (Figure 6C). HFD mice had significantly higher urinary excretion of the oxidative stress marker, hydrogen peroxide (H_2_O_2_), during the dark phase compared with the light phase (Figure 6D). Urinary excretion of the DNA damage product 8-hydroxy 2-deoxyguanosine (8-OHdG) was significantly reduced by TRF in HFD mice during the dark phase (Figure 6E). TRF did not influence diurnal water intake, urine output, or electrolyte excretion by HFD mice (Supplemental Table 3).

### TRF alters rhythms of plasma hormones, metabolites, and adipokines.

We performed additional analyses of circulating factors known to be important contributors to obesity-dependent cardiometabolic dysfunction, utilizing a cohort of mice with blood collections every 4 hours. Insulin-like growth factor 1 (IGF-1) is a liver-derived hormone with insulin-like effects (37) and affects vascular function. Plasma IGF-1 levels were arrhythmic in ad libitum ND and HFD mice, whereas TRF mice showed rhythmicity (Supplemental Figure 3A and Supplemental Table 4). IGF-1 rhythms in TRF mice on HFD and ND were antiphase to each other, with HFD+TRF peaking in the light phase and ND+TRF peaking in the dark phase (Supplemental Table 4). The ketone body metabolite, beta hydroxybutyrate (BHB), is mainly derived from fatty acids in the liver and is important for energy metabolism in extrahepatic tissues (38). Circulating levels of BHB were not significantly different between groups and were higher during the dark phase (Supplemental Figure 3B and Supplemental Table 4). Plasma BHB levels were rhythmic in all diet groups except the ND+TRF group. HFD shifted the phase of BHB compared with ND, but rhythms were similar in HFD and HFD+TRF mice (Supplemental Table 4). Nonesterified fatty acids (NEFAs) are metabolites that are positively associated with vascular and organ damage in models of diet-induced obesity (11). Plasma NEFA concentrations were rhythmic in ND, ND+TRF, and HFD+TRF mice but were arrhythmic in ad libitum HFD mice (Supplemental Figure 3C and Supplemental Table 4). TRF significantly shifted the NEFA phase in ND mice (Supplemental Table 4).

Plasma concentration of nitrite (an NO metabolite) has been shown previously to reflect endothelial function acutely (19–21, 39, 40). In contrast, plasma nitrate levels are known to reflect dietary nitrate intake (41, 42). Thus, we measured plasma nitrite and nitrate levels from a cohort of mice with blood collections every 4 hours. Plasma nitrite was arrhythmic in all groups of mice, while plasma nitrate was rhythmic in ND+TRF and HFD+TRF mice (Supplemental Figure 3, D and E, and Supplemental Table 4).

We measured markers of kidney function in plasma at 4-hour intervals over a 24-hour period. The results (Supplemental Figure 3, F and G, and Supplemental Table 5) revealed that blood urea nitrogen (BUN) levels were similar across the 4 dietary and feeding groups and were arrhythmic in all groups of mice. Plasma creatinine levels were also similar among all 4 dietary and feeding groups but rhythmic only in HFD mice.

Adipokines (adipose-derived factors such as adiponectin and leptin) have key metabolic roles and were previously shown to have direct effects on the vasculature, particularly on the endothelium (43–51). Plasma adiponectin concentration was unchanged across all groups during the light and dark phase, although a time of day × time of feeding interaction was significant (Supplemental Figure 4A). Plasma leptin levels were significantly higher in ad libitum HFD and HFD+TRF mice compared with ND mice at ZT1, ZT5, ZT12, and ZT17 while TRF significantly decreased leptin only at ZT12 in HFD mice (Supplemental Figure 4B). There were significant main effects of diet, time of feeding, and time of day as well as significant interactions of time of day × diet and diet × time of feeding.

Plasma levels of the lipid peroxidation biomarker 8-isoprostane were measured to assess oxidative stress in HFD and HFD+TRF mice during the light (ZT5) and dark (ZT12) phase. TRF trended to reduce 8-isoprostane in HFD mice at ZT5 compared with HFD+TRF mice at ZT12 (Supplemental Figure 5A); however, the DNA damage product 8-OHdG was not affected by time of feeding or time of day in plasma from HFD mice (Supplemental Figure 5B).

## Discussion

Here we show evidence that a short-term TRF intervention reduces cardiovascular disease risk in a mouse model of established diet-induced obesity. The major findings of this study demonstrate that 2 weeks of TRF in an established model of diet-induced obesity (a) do not reduce body weight; (b) improve whole-body energy metabolic rhythms; (c) restore BP and HR dipping; (d) restore aortic PWV, and reduce aortic wall thickness, improving aortic health; and (e) abrogate renal proximal tubular injury and reduce kidney medullary fibrosis and collagen content, as well as kidney T cell infiltration, improving kidney health. These exciting results complement clinical data suggesting significant TRF-induced benefits to reduce cardiovascular disease risk factors in obesity and that TRF may be an effective intervention for improving health in obesity.

### TRF, cardiometabolic rhythms, and BP rhythms.

We examined whole-body metabolic rhythms in this obesity model and found that ad libitum consumption of 45% HFD abolished circadian changes in the RER compared with mice fed an ND. Our findings show TRF improved RER light versus dark difference in both ND and HFD mice, similar to a previous study in which mice were fed a 61% fat HFD and subjected to dark-phase 8-hour TRF for at least 12 weeks (31). Previous studies in mice on a chronic ≥60% HFD, with TRF maintained throughout the duration of HFD feeding for 20 weeks, showed that TRF protected against hepatic steatosis and reduced insulin resistance as well as altered glucose metabolism and lipid homeostasis (32). The current study demonstrates that 2 weeks of TRF intervention is sufficient to improve whole-body metabolic diurnal rhythms in mice that had been fed an HFD ad libitum for a prolonged (20-week) period.

Normal BP and HR rhythms show a decrease or dip during the light phase in rodents. In the present study, we found that this chronic HFD model leads to markedly reduced dipping of BP and HR compared with ND mice, with TRF restoring BP and HR dipping in HFD mice to levels similar to those of ND mice. Prior studies of mice fed a 60% HFD for periods ranging from 8 weeks to 16 months found a modest increase or no change in BP and/or HR (52–55). In another model characterized by obesity, the leptin receptor–deficient *db/db* mouse, BP dipping was reduced along with an elevated 24-hour BP (56, 57). Type II diabetes in humans is also associated with disrupted diurnal BP variation (58, 59). Our analysis of HR variability suggests that TRF in HFD mice may improve parasympathetic tone during the inactive/fasting light period, which is linked to restoration of BP and HR dipping. Importantly, it is well documented that a reduced BP dipping profile increases the risk for hypertension, end-organ damage, and cardiovascular disease in humans (15, 60). Our findings indicate that the feeding/fasting cycle is a critical mediator in systemic cardiovascular rhythms.

### TRF and aortic health.

Aortic damage, such as increased wall thickness and fibrosis, and aortic stiffness are independent risk factors of hypertension and cardiovascular disease (61, 62). Our focus was to determine whether TRF would mitigate HFD-induced aortic damage and stiffness. As expected, we found that HFD mice had increased aortic medial hypertrophy, or wall thickness, and increased aortic stiffness measured by PWV compared with ND mice. HFD mice exposed to short-term TRF displayed an aortic wall thickness and aortic stiffness measure similar to that seen in ND mice. It is established that the loss of NO bioavailability is linked to aortic damage and stiffness as well as smooth muscle reprogramming (63, 64). However, at this point, the TRF-dependent mechanism of reversing aortic remodeling is unclear.

Several studies have demonstrated endothelial dysfunction in rodent models of diet-induced obesity (19, 22, 23). Most studies reported in the literature analyzed endothelial function during the light phase in rodent models, and previous studies with chronic HFD did not determine time-of-day changes in endothelial function (19, 21, 53, 55). Our study assessing vascular reactivity during both light and dark phases revealed that HFD mice display aortic endothelial dysfunction only during the light phase and that TRF reduces the endothelial dysfunction. Endothelial dysfunction is a mediator of the loss of vascular health (64) and contributes to aortic hypertrophy, fibrosis, and inflammation (65, 66).

Oxidative stress, including increased reactive oxygen species, is an established cause of reduced NO bioavailability leading to endothelial dysfunction in the aorta (67). Isoprostanes, markers of oxidative stress and lipid peroxidation, also contribute to cardiovascular disease (68). A recent study reported 5 weeks of TRF decreased 8-isoprostane levels in prediabetic, overweight men and was associated with lower BP (24). Our data in obese male mice suggest that TRF tends to reduce 8-isoprostane in association with restored aortic endothelial function and BP dipping in the light period, possibly mediated by reducing oxidative stress, although further research is warranted to decipher this mechanism. HFD mice also had increased aortic stiffness that was normalized by TRF, perhaps due to TRF-mediated decreased oxidative stress. Further research is needed to determine the TRF-induced molecular mechanistic regulation of decreasing oxidative stress.

Previous reports show that plasma nitrite levels are indicative of acute NO activation and that nitrite and nitrate levels have diurnal variation; however, time-dependent changes in these parameters in animals chronically fed an HFD remained speculative (69). Surprisingly, plasma nitrite concentrations were not rhythmic in any of our groups of mice, suggesting that this is not a robust marker of HFD- or TRF-induced changes in endothelial function. Dietary nitrate contributes to the levels of plasma nitrate (70, 71), and our observation that TRF institutes a rhythm in plasma nitrate in both ND and HFD suggests that the timing of food intake affects the level of circulating nitrate.

Endothelial function and vascular contractility show time of day–dependent changes under normal dietary conditions with endothelial function lowest when vasoconstriction is highest at the beginning of the active period (16–18). Our study validates previous reports of a significant time-of-day difference in the total response to adrenergic vasoconstriction in mice fed an ND, with a greater constriction of aortic rings isolated during the dark phase (16, 72). In our study with 45% HFD mice, vasoconstrictor sensitivity of aortic rings to an adrenergic agonist was significantly greater in the dark phase compared with the light phase. Interestingly, TRF increased the sensitivity to adrenergic vasoconstrictor stimuli in HFD mice during the light phase, similar to that seen in the dark phase. Previous reports utilizing mice on a chronic 60% HFD demonstrated blunted aortic vasoconstriction that presumably was assessed during the light phase, although time of day was not specifically stated in the publication (53, 55). Vascular adrenergic activation is a balance of direct stimulation of the smooth muscle adrenergic receptor–mediated vasoconstriction and an indirect endothelial alpha 2– and beta-adrenergic receptor–stimulated NO release, with oxidative stress directly influencing this equilibrium (73). At this point, the mechanism for the TRF-induced increased sensitivity to adrenergic stimulation in HFD mice is unclear.

### TRF and kidney health.

Kidney injury has been observed in obese patients with normal renal function (74), and obesity is a major risk factor for chronic kidney disease and various cardiovascular disorders. We did not observe differences in plasma creatinine or BUN as measures of kidney function in our model. However, the present study provides data showing that TRF mitigates many of the HFD-induced kidney damage assessments. TRF restored the proximal tubular brush border integrity. Proximal tubules are high-energy-demanding cells, especially under high-fat conditions with increased fatty acid oxidation (75). Previous reports show that HFD promotes proximal tubular damage via oxidative stress (11). TRF prevented the dark-phase increase in excretion of the urinary oxidative stress marker 8-OHdG, suggesting that TRF in HFD mice reduces oxidative stress in the kidney. Thus, we propose that TRF may protect against proximal tubular damage via reducing oxidative stress.

Kidney fibrosis is one of the hallmarks of end-stage kidney disease and HFD-induced kidney damage (11, 76–78). We found that TRF reversed the HFD-induced medullary vasa recta fibrosis, as well as abolishing the HFD-induced increase in medullary type I and III collagen fibers. In contrast, HFD-induced glomerulosclerosis was unchanged by TRF. The distinct mechanisms of HFD-induced glomerulosclerosis and medullary vasa recta fibrosis are unclear at this point. Endothelial cells in the kidney medullary vasa recta are highly specialized and distinct from endothelial cells of the glomeruli and large arteries, both of which respond to environmental stimuli via disparate mechanisms (79).

HFD-induced kidney damage is also mediated by the activation and/or infiltration of immune cells, such as macrophages and T cells (52, 77, 80). We saw no difference in the numbers of macrophages (F4/80^+^) in the 4 groups of mice, although specific subtypes of macrophages and the activation status were not determined. In contrast, TRF reduced the HFD-induced increase in T cell (CD3^+^) numbers within the renal medullary vasa recta region associated with the amelioration of fibrosis in the same region. We propose that TRF blocks the increased T cell infiltration or endothelium dysfunction in the kidney medullary vasa recta. Future studies are needed to determine the specific subtypes of immune cells responsive to TRF and potential mechanisms of T cell infiltration.

We examined urinary markers of kidney injury in all 4 groups of mice. Urine albumin is a marker of kidney glomerular injury used in many investigations. Our model of HFD did not increase albumin or KIM-1 excretion compared to mice on ND, although we found greater glomerulosclerosis and loss of brush border integrity. NGAL excretion is utilized as a biomarker of the severity of kidney injury as it is released and excreted by injured or inflamed kidney tubular cells and immune cells (80, 81). This obesity model also did not show an increase in NGAL excretion compared to ND control mice. However, there was a significant day/night difference in NGAL excretion in HFD mice. These urinary markers of kidney damage appear not to be reflective of the histological changes observed in this obesity model. We propose that further research is needed to decipher obesity-specific urinary kidney damage markers.

Our model employing chronic (20 weeks) feeding of 45% HFD verified that the metabolic mediators, IGF-1, BHB, NEFA, leptin, and adiponectin, show time-dependent changes. A prior study showed that metabolic mediators, including NEFA and leptin, display disrupted rhythms in mice fed 6 weeks of a chronic 45% HFD (7). IGF-1 levels are lower in models of HFD and IGF-1 supplementation offers cardioprotection (82, 83). We further observed that timing of feeding/fasting did not substantially change rhythms of IGF-1 and NEFA predominantly in ND mice. While plasma BHB showed diurnal variation, diet or timing of feeding did not significantly change rhythms. Recently, BHB was shown to lower BP in a model of salt-sensitive hypertension (84). Fasting or reduced caloric intake increases BHB, and humans with obesity-related nonalcoholic fatty liver disease have lower BHB (85, 86). We also observed that leptin levels in HFD mice were increased throughout the light phase and reduced by TRF during the early dark phase. Hatori et al. (31) reported that long-term TRF in mice on a 61% HFD lowered leptin following glucose administration or overnight fasting. Leptin is known to have pleiotropic effects regulating cellular metabolism and vascular function, including direct effects on the endothelium, smooth muscle, and immune cells (43–48). Leptin resistance or leptin signaling deficiency observed in obesity leads to increased cardiovascular disease risk (87) through a plethora of mechanisms. Further investigation is needed to determine whether leptin signaling is directly responsible for the TRF-induced changes in BP, HR, aortic function, and organ damage observed in this study.

In conclusion, the present study demonstrates that a short-term TRF intervention following an established chronic ad libitum HFD protocol improves vascular and kidney health. These findings underscore the importance of maintaining behaviors that align with physiological rhythms for maintaining cardiovascular health (88–93), especially in obesity. We anticipate that there is not a sole mechanism driving the TRF-mediated improvements in vascular and kidney health in obesity. These studies provide a wide range of potentially novel and impactful discoveries related to TRF as an intervention in obesity to reduce cardiovascular disease risk factors.

## Methods

### Sex as a biological variable.

Male C57BL/6J mice (Jackson Laboratory) were utilized in this study. Our study exclusively examined male mice in this model of diet-induced obesity. It is unknown whether the findings are relevant for female mice.

### Animals.

Male 6-week-old C57BL/6J mice (Jackson Laboratory) were provided standard laboratory chow and water ad libitum and kept on a 12-hour light/12-hour dark cycle (ZT0 = lights on, 7 am; ZT12 = lights off, 7 pm) in a temperature- and humidity-controlled environment in standard cages that are called “home cages.” Mice were acclimated to this environment for 2 weeks before the diet protocols started.

### Diet-induced obesity and the TRF intervention protocol.

Beginning at 8 weeks of age, male mice were fed ad libitum either an ND (10% fat, 3.85 kcal/g; Research Diets, catalog D12450K) or an HFD (45% fat, 4.73 kcal/g; Research Diets, catalog D12451) for 18 weeks. ND and HFD groups were then subjected to TRF or sham intervention for 2 weeks of feeding (from week 18 to week 20). TRF protocol involved research personnel removing food containers to empty the food and replacing empty food containers for 12 hours during the dark phase (between ZT12 and ZT0), while the sham protocol involved research personnel removing the food containers and replacing the same containers with replete food. All mice were exposed to the food containers moving in and out. These mice are denoted as ND, ND+TRF or HFD, HFD+TRF throughout the study. Mice were group-housed (3 mice/cage) in home cages for the majority of the measurements. Body weight was monitored weekly throughout the feeding protocols in their home cages. Food intake was monitored weekly throughout the protocols and normalized per mouse, with food or caloric intake data reported as g/d and kcal/d. Separate cohorts of mice implanted with telemetry devices (Data Sciences International) were single-housed in standard home cages with TRF proceeding as stated above. CLAMS and metabolic cage studies were performed with separate cohorts of mice that were single-housed in the requisite specialized cages (Columbus Instruments). These mice were acclimated to the cages for 7 days or 3 days prior to the data collection periods, respectively. Mice in specialty cages also proceeded with ad libitum or TRF protocols.

### Statistics.

GraphPad Prism 9 or IBM SPSS Statistics 26 was used for statistical analysis. Data are represented as mean ± SEM, and statistical significance was set at *P* < 0.05. Figure legends and table legends indicate specific statistical tests used for each dataset. Three-way repeated measures ANOVA was used to compare light- and dark-phase differences in RER, BP, HR, activity, food intake, water intake, urine production, urinary Na^+^ excretion, and renal damage markers. Two-way ANCOVA was used for RMR calculated from EE. Body weight, fat mass, lean mass, aortic PWV, quantitative PCR, and histology data were analyzed with 2-way ANOVA with Tukey’s post hoc test. For telemetry and vascular reactivity data at multiple time points, comparisons were made with 3-way ANOVA by Holm-Šídák post hoc test. Two-way ANOVA was used to compare diet and time of feeding during the light or dark period for telemetry and vascular reactivity data. Cosinor analysis of telemetry data was used to assess rhythmic and circadian variables in BP, HR, and locomotor activity (94). Cosinor analysis was used to analyze diurnal changes in plasma metabolites, BUN, and creatinine (95). Three-way ANOVA was used to compare plasma adipokine measurements at 4 time points.

### Study approval.

All animal procedures were approved by the Institutional Animal Care and Use Committee at the University of Alabama at Birmingham (UAB) and were compliant with the NIH *Guide for the Care and Use of Laboratory Animals* (8th ed., National Academy of Sciences, 2011).

### Data availability.

Data from this paper are provided in the main text or the Supporting Data Values file in the supplement, or requests for specific data files should be directed toward the corresponding author.

Further information can be found in Supplemental Methods.

## Author contributions

PP, CDM, DZ, SMB, KLG, DMP, and JSP conceived and designed research; PP, CDM, DZ, JC, CJE, LSD, TMS, JMA, SNB, YZ, and RS performed experiments; PP, DZ, JRP, CDM, JMA, CJE, MKR, LSD, RS, SMB, KLG, DMP, and JSP analyzed data; PP, DZ, JRP, LSD, CDM, MKR, DMP, SMB, KLG, DMP, and JSP interpreted results of experiments; PP and CDM prepared figures; PP and CDM drafted the manuscript; PP, JRP, CDM, LSD, DMP, SMB, KLG, and JSP edited and revised the manuscript; and all authors approved the final submission. PP and CDM made equal contributions to this research and are co–first authors. PP initiated the study and was identified to be listed first.

## Supplementary Material

Supplemental data

Supporting data values

## Figures and Tables

**Table 1:**
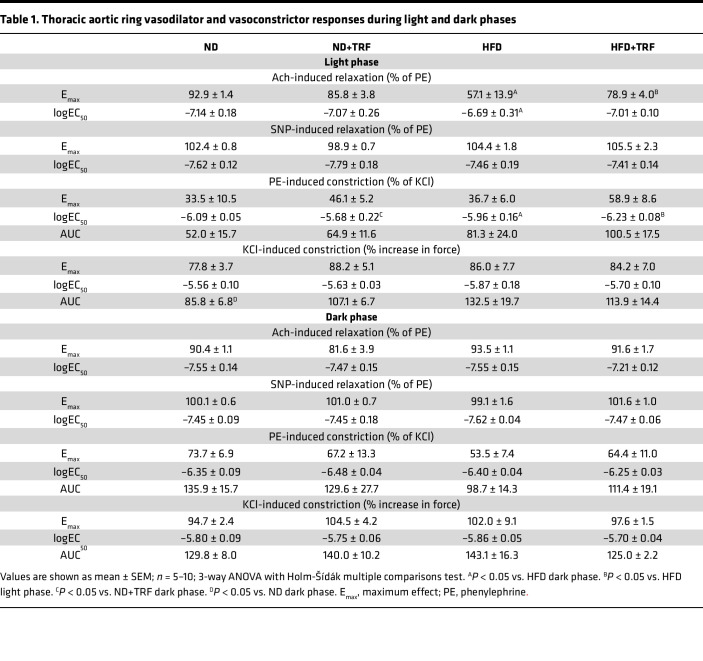
Thoracic aortic ring vasodilator and vasoconstrictor responses during light and dark phases

## Version 1 01/15/2025

In-Press Preview

## Version 2 02/24/2025

Electronic publication
