# Supplementary material for: Time-restricted feeding reduces cardiovascular disease risk in obese mice
Source: JCI Insight. 2025 Jan 7;10(4):e160257. doi: 10.1172/jci.insight.160257 (PMC11949066; doi:10.1172/jci.insight.160257)
Supplement: Supplemental data [file jciinsight-10-160257-s151.pdf]

SUPPLEMENT

Time-restricted feeding reduces cardiovascular disease risk in mice with diet-induced obesity

Paramita Pati, [*1] **Carmen De Miguel**,[*1] Jodi R. Paul,[2] Dingguo Zhang,[1] Jackson Colson,[1] John Miller Allan,[1] Claudia J. Edell,[1] Megan K. Rhoads,[1] Luke S. Dunaway,[1] Sara N. Biswal,[1] Yihan Zhong,[1] Randee Sedaka,[1] Telisha Millender-Swain,[3] Shannon M. Bailey,[3] Karen L. Gamble,[2] David M. Pollock,[1] and Jennifer S. Pollock[1]

[1]Cardio-Renal Physiology & Medicine, Division of Nephrology, Department of Medicine
[2]Division of Behavioral Neurobiology, Department of Psychiatry
[3]Division of Molecular and Cellular Pathology, Department of Pathology
Heersink School of Medicine, University of Alabama at Birmingham, Birmingham, AL

*Denotes co-first authors

Supplemental Methods, pages 2-5
Supplemental Tables, pages 6-10
Supplemental Figures, pages 11-16

Supplemental Methods

Sex as a biological variable. Male C57BL/6J mice (Jackson Laboratory, Bar Harbor, ME) were utilized in this study. Our study exclusively examined male mice in this model of diet-induced obesity. It is unknown whether the findings are relevant for female mice.

Animals. Six-week-old male C57BL/6J mice (Jackson Laboratory, Bar Harbor, ME) were provided standard laboratory chow and water ad libitum and kept on a 12 h light:12 h dark cycle (ZT0 = lights on, 7 a.m.; ZT12 = lights off, 7 p.m.) in a temperature and humidity-controlled environment in standard cages that are called "home cages". Mice were acclimated to this environment for 2-wk before the diet protocols started.

Diet-induced obesity and the TRF intervention protocol. Beginning at 8 weeks of age, male mice were fed *ad libitum* either a normal diet (ND; 10% fat, 3.85 kcal/g; Research Diets, New Brunswick, NJ, catalog #D12450K) or a HFD (45% fat, 4.73 kcal/g; Research Diets, New Brunswick, NJ, catalog #D12451) for 18 weeks. ND and HFD groups were then subjected to TRF or sham intervention for 2 weeks of feeding (from week 18 to week 20). TRF protocol involved research personnel removing food containers to empty the food and replacing empty food containers for 12 h during the dark phase (between ZT12 and ZT0), while the sham protocol involved research personnel removing the food containers and replacing the same containers with replete food. All mice are exposed to the food containers moving in and out. These mice are denoted as ND, ND+TRF or HFD, HFD+TRF throughout the study. Mice were group housed (3 mice/cage) in home cages for the majority of the measurements. Body weight was monitored weekly throughout the feeding protocols in their home cages. Food intake was monitored weekly throughout the protocols and normalized to per mouse with food or caloric intake data reported as g/day and kcal/day. Separate cohorts of mice implanted with telemetry devices were single housed in standard, home cages with TRF proceeding as stated above. CLAMS and metabolic cage studies were performed with separate cohorts of mice that are single housed in the requisite specialized cages. These mice were acclimated to the cages for 7 days or 3 days prior to the data collection periods, respectively. Mice in specialty cages also proceeded with ad lib or TRF protocols.

Indirect calorimetry. Continuous 24 h monitoring of metabolic parameters was performed using Comprehensive Lab Animal Monitoring System (CLAMS, Columbus Instruments Inc., Columbus, OH, USA) for indirect calorimetry in a subset of mice. Mice were single housed and acclimated to the CLAMS for 7 days before initiating measurements. ND or HFD as well as TRF or sham intervention feeding were maintained while mice were housed in the CLAMS. Respiratory exchange ratio (RER) and energy expenditure (EE) over 24-hr were assessed as previously reported (95, 96). CLAMS data collected during week 20 of the protocol were analyzed across 4 days using batch analysis and activity profile functions in ClockLab software (Actimetrics, Wilmette, IL, USA). Energy expenditure was normalized to lean body mass (97). Resting metabolic rate (RMR) was determined from EE during the lowest 2-h period in the light phase.

Quantitative magnetic resonance. To assess body composition, fat and lean mass were measured in conscious mice by quantitative magnetic resonance (QMR; Echo 3-in-1, Echo Medical System) at the UAB Nutrition and Obesity Research Center Core facility as described previously (96, 98).

Telemetry measurements. Telemetry was utilized to monitor BP, HR, and locomotor activity. Telemetry mice were implanted with transmitters during the ad libitum ND or HFD feeding protocol. At the time of surgical implantation, mice were 12-16 weeks of age and had *ad libitum* ND or HFD for 4-8 weeks. Mice were anesthetized with isoflurane and implanted with an arterial catheter into the right carotid artery connected to a transmitter (PA-C10; Data Sciences International, St. Paul, MN) to continuously monitor arterial pressure and HR as previously described (99-102). Mice were allowed to

fully recover from surgery prior to telemetry data collection. Data were collected during the final week of the 20-wk study. Baseline recordings of SBP, DBP, MAP, HR, and locomotor activity were collected every 10 min using the DataQuest System (Data Sciences International, St. Paul, MN) and analyzed using Ponemah v6.3 (DSI, Minneapolis, MN). The average of the final three consecutive days of continuous 24-h data collection was calculated for each animal. Anticipatory activity was determined by calculating the amount of activity occurring from ZT 8-12 (as percent of total daily activity). Blood pressure waveforms were obtained from telemeter recordings using DSI Ponemah v6.4 software, and HR variability analysis was conducted in the same software suite using inter-beat intervals with maximum slope as the marker. Blood pressure waveforms were viewed manually to confirm markers, and periods of noise or artifacts were excluded from the analysis. Values > 3 standard deviations from the mean were removed as outliers. Data collected during ZT3–ZT9 were analyzed for the light phase and ZT15–ZT21 for the dark phase.

Metabolic cage studies. Food and water intake and urine output were measured in 12-h increments using metabolic cages. Mice were acclimated to cages for 2 days before food and water intake and urine volume were measured gravimetrically using a Mettler Toledo NewClassic SG analytical balance (ML1502E). Research personnel determined initial and final weights of food containers, water bottles, and urine containers from light (ZT0–ZT12) and dark (ZT12–ZT0) phases gravimetrically. ND or HFD as well as TRF or sham intervention feeding were maintained while mice were housed in the metabolic cages. Light and dark phase urine samples were stored at –80°C until performing assays.

Urine assays. Urinary sodium and potassium concentrations were determined by atomic absorption spectrometry (Analyst 200, Perkin Elmer, Waltham, MA) (101, 103). Urinary renal injury markers were measured using ELISA kits for mouse neutrophil gelatinase-associated lipocalin (NGAL) (Abcam, Cambridge, MA, catalog # ab119601, assay range 78–5000 pg/mL) and kidney injury molecule-1 (KIM-1) (Abcam, Cambridge, MA, catalog # ab119596, 31.2–2000 pg/mL). Urinary albumin concentration was determined by ELISA (GenWay Biotech Inc., San Diego, CA, catalog # GWB-282C17, 7.8–500 ng/mL). Urinary hydrogen peroxide (H_2O_2) concentration was measured using an amplex red assay (Invitrogen, Waltham, MA, catalog # A22188, 0–10 μM), and urinary 8-OHdG levels were measured by ELISA (Abcam, Waltham, MA, catalog # ab201734, 0.94 ng/mL – 60 ng/mL). Each assay was conducted per the manufacturer's instructions and values were confirmed to be within the linear portion of the standard curve.

Aortic vascular reactivity. Thoracic aortae were prepared for vascular reactivity experiments at ZT0 and ZT12 as previously described (102, 104). Briefly, 2 mm thoracic aortic segments were dissected in ice-cold physiological salt solution and perivascular adipose tissue was removed as described previously (105, 106). Vascular function was assessed using the Multi Wire Myograph System - 620M (DMT-USA, Inc., Ann Arbor, MI) with LabChart 8 software (ADInstruments, Colorado Springs, CO). Aortic rings were allowed to equilibrate for 30 min in warmed (37°C), gassed (95% O_2:5% CO_2) physiological saline solution (107). Passive tension was set to 10 mN for aortic segments. Cumulative concentration–response curves to acetylcholine (Ach; 1×10^{-9} to 10^{-5} mol/L) were performed after preconstriction of aortic segments with phenylephrine (PE). Cumulative concentration–response curves were also performed with sodium nitroprusside (SNP; 1×10^{-10} to 10^{-5} mol/L). To assess vasoconstriction, cumulative concentration responses to PE were generated and normalized to the maximum constriction elicited by 100 mmol/L potassium chloride (KCl). Data are presented as maximum relaxation (E_{max}) as a percentage of PE and sensitivity as $logEC_{50}$ during the light or dark periods. Area under the curve (AUC) was calculated for PE and KCl concentration response curves during the light or dark periods. Since the vascular reactivity protocol requires 6+ hours to complete, the results refer to the measurements as "light" or "dark".

Aortic pulse wave velocity (PWV). Aortic stiffness at ZT2–ZT5 was assessed based on PWV measured with a Vevo 3100 ultrasound Doppler probe at the UAB Mouse Cardiovascular Phenotyping

Core. Mice were anesthetized with isoflurane and placed in a supine position for ECG measurements at the proximal and distal aorta. Aortic PWV was calculated by dividing the distance between measurement sites by the time difference between pulse arrivals as determined by ECG R-peaks.

Aorta pathology. Thoracic aortae were collected at ZT17 and placed in a 10% buffered formalin solution. Formalin-fixed tissues were embedded in paraffin, sectioned at 5 µm thickness, and stained with H&E, Masson's Trichrome, and Picrosirius red. Aortic wall thickness was measured as the difference between the external elastic lamina and the internal elastic lamina (CellSens software, Olympus US, Center Valley, PA). Aorta fibrosis was quantified using Metamorph software (Molecular Devices, San Jose, CA) (108, 109). Picrosirus red-stained aortic sections were examined under polarized light to assess collagen content (Metamorph).

Kidney pathology. Kidneys from a separate set of mice were collected at ZT12 and placed in a 10% buffered formalin solution. Formalin-fixed tissues were embedded in paraffin and sectioned at 4 µm thickness. Sections were stained with Masson's trichrome, Picrosirius red, and Periodic Acid Schiff Hematoxylin (PASH) staining for renal damage analysis. Picrosirus red-stained kidney sections were examined under brightfield and polarized light to assess collagen type I and III content (Metamorph). Kidney sections were also stained with primary antibodies specific for CD3 (Abcam, Cambridge, MA, catalog # ab16669) and F4/80 (BioRad, Hercules, CA, catalog # MCA497GA) as described previously (110, 111). Histological scoring of renal damage as well as quantitation of $CD3^+$ and $F4/80^+$ cells were performed in a blinded manner as described previously (110, 111).

Quantitative RT-PCR. RNA was extracted from mouse outer medulla samples from ZT13 using Invitrogen PureLink RNA Mini kit (Invitrogen, *Waltham, MA*) and quantified by spectrophotometric analysis (NanoDrop ND-1000, Thermo Scientific, Waltham, MA). Reverse transcription to cDNA was performed with Quantitect Reverse Transcription kit (Qiagen, Valencia, CA) following instructions provided by the manufacturer. Primers for fibronectin 1 (*Fn1*) were synthesized by Integrated DNA Technologies (IDT, Coralville, IA). Primer sequences were as follow: *Forward*: 5' GATGTCCGAACAGCTATTTACCA 3'; *Reverse:* 5' CCTTGCGACTTCAGCCACT 3' (NM_001276408). Ribosomal protein L13a (*Rpl13a)* was used as housekeeping gene (Qiagen, QT00267197, NM_009438). RNA expression was detected with Quantitect SYBR green kit (Qiagen) and using a CFX96 Touch RT-PCR detection system (Bio-Rad, Hercules, CA).

Plasma assays. Mice were anesthetized with isoflurane and cardiac puncture was used for blood collection.[43] Plasma was separated by centrifugation at 2,000 *g* for 15 min, snap frozen in liquid nitrogen, and stored at -80°C until thawed for assays. To determine the influence of TRF on oxidative stress in HFD mice, we utilized cohorts of HFD and HFD+TRF mice with plasma collected at ZT5 and ZT12. Plasma 8-iso-prostaglandin $F_{2\alpha}$ (8-isoprostane) was measured using a direct ELISA kit (Enzo Life Sciences, Farmingdale, NY, catalog # ADI-900-091, 160 - 100,000 pg/mL). For the 8-isoprostane assay, plasma (160 µL) was hydrolyzed with 40 µL 10N NaOH, neutralized with 40 µL 12.1N HCl, and diluted (1:4.5). Plasma 8-OHdG concentration was measured using an ELISA kit (Abcam, Waltham, MA, catalog # ab201734, 0.94 ng/mL - 60 ng/mL). To determine rhythms of plasma nitrite and nitrate, we utilized a cohort of ND, ND+TRF, HFD, and HFD+TRF mice with plasma collected at 4-h intervals over a 24-h period as described previously (99, 112). Plasma nitrite and nitrate were measured by high performance liquid chromatography (ENO-30, Eicom, Kyoto, Japan, 0-400 pmol)(101, 113) with both reported as µM. The hormone insulin-like growth factor 1 (IGF1) was measured in plasma using an ELISA kit (R&D Systems, Minneapolis, MN, catalog # MG100, 31.2 - 2,000 pg/mL). Plasma ketone body and lipid levels were measured using enzymatic assay kits for beta hydroxybutyrate (BHB) (Cayman Chemical, Ann Arbor, MI, catalog # 700190, 0-0.5 mM) and non-esterified fatty acids/free fatty acids (NEFA/FFA) (ZenBio, Research Triangle Park, NC, catalog# SFA-1, 0-1000 µM). Plasma blood urea nitrogen (BUN) was measured with a colorimetric assay (Abcam, Waltham, MA, catalog # EIABUN, 0.156–10 mg/dL). Plasma creatinine was measured by isotope dilution LC-MS/MS at the UAB O'Brien Center Core. In a separate cohort of mice, plasma adipokines, adiponectin (R&D Systems,

Minneapolis, MN, catalog # MRP300, 0.2 - 10 ng/mL), and leptin (Crystal Chem, Elk Grove Village, IL, catalog # 90030, 0.2 - 12.8 ng/mL) were measured using ELISA kits. Assays were conducted per the manufacturer's instructions and values were confirmed to be within the linear portion of the standard curve.

Statistics. GraphPad Prism 9 (GraphPad Software Inc., La Jolla, CA) or IBM SPSS Statistics 26 (IBM Corp., Armonk, NY) were used for statistical analysis. Data are represented as mean $\pm$ SEM and statistical significance was set at $P<0.05$. Figure legends and table legends indicate specific statistical tests used for each data set. Three-way repeated measures ANOVA was used to compare light and dark phase differences in RER, BP, HR, activity, food intake, water intake, urine production, urinary Na^+ excretion and renal damage markers. Two-way ANCOVA was used for RMR calculated from EE. Body weight, fat mass, lean mass, aortic PWV, QPCR, and histology data were analyzed with two-way ANOVA with Tukey's post hoc test. For telemetry and vascular reactivity data at multiple time points, comparisons were made with three-way ANOVA by Sidak's post hoc test. Two-way ANOVA was used to compare diet and time of feeding during the light or dark period for telemetry and vascular reactivity data. Cosinor analysis of telemetry data was used to assess rhythmic and circadian variables in blood pressure, heart rate, and locomotor activity (117). Cosinor analysis was used to analyze diurnal changes in plasma metabolites, BUN, and creatinine (114). Three-way ANOVA was used to compare plasma adipokine measurements at 4 time points.

Study approval. All animal procedures were approved by the Institutional Animal Care and Use Committee at the University of Alabama at Birmingham and were compliant with the National Institutes of Health *Guide for the Care and Use of Laboratory Animals* (8th ed., National Academy of Sciences, 2011).

Data availability. Data from this paper are provided in the main text or the Supporting Data Values file in the supplement or requests for specific data files can be directed towards the corresponding author.

Supplemental Table 1. Circadian analysis of telemetry data

Parameter	**Mesor**	**Amplitude**	**Acrophase**
		ND	
Systolic blood pressure	129±1	22.9±1.0*	18.7±0.1
Diastolic blood pressure	94±1	18.3±0.9*	18.5±0.2
Mean arterial pressure	112±1	20.4±0.9*	18.5±0.2
Heart rate	564±9	10.9±0.8*	16.4±0.4
Locomotor activity	5.6±0.7*	8.3±1.2*	17.8±0.2
		ND+RF	
Systolic blood pressure	125±5	27.6±0.9	18.6±0.1
Diastolic blood pressure	94±2	23.6±1.2	18.5±0.1
Mean arterial pressure	110±3	25.7±1.0	18.5±0.1
Heart rate	571±16	14.2±1.0	16.8±0.2
Locomotor activity	7.9±0.5	13.2±1.5	17.8±0.3
		HFD	
Systolic blood pressure	135±3	21.4±1.1†	18.7±0.2
Diastolic blood pressure	97±2	15.2±0.8†	18.6±0.2
Mean arterial pressure	117±2	18.4±0.9†	18.6±0.2
Heart rate	602±7	96.7±8.7†	16.8±0.1
Locomotor activity	4.2±0.4	5.4±0.7	18.0±0.3
		HFD+RF	
Systolic blood pressure	130±2	25.5±1.4	19.0±0.1
Diastolic blood pressure	92±3	18.9±1.0	19.0±0.1
Mean arterial pressure	112±3	22.5±1.1	18.9±0.1
Heart rate	574±5	14.5±0.9	17.4±0.2
Locomotor activity	4.7±0.2	5.8±0.6	18.3±0.3

Values are ± SE; n=6-8

*P<0.05 ND vs. ND+RF

†P<0.05 HFD vs. HFD+RF

MAP, Amplitude: Diet: p=0.0144, Time of feeding p<0.0001, Interaction: ns
SBP, Amplitude: Diet: ns, Time of feeding p=0.0004, Interaction: ns
DBP, Amplitude: Diet p=0.0006, Time of feeding p=0.0001, Interaction: ns
HR, Mesor: Diet: p=0.0481, Time of feeding: ns, Interaction: ns
HR, Amplitude: Diet: ns, Time of feeding p=0.0001, Interaction: ns
Activity, Mesor: Diet p=0.0001, Time of feeding p=0.0089, Interaction: ns
Activity, Amplitude: Diet p<0.0001, Time of feeding p=0.0197, Interaction: p=0.0492

Supplemental Table 2. Time domain measures of heart rate variability

ND	ND+TRF	HFD	HFD+TRF
**Light phase**			
N-N Interval (msec)			
120.1 ± 3.6	128.7 ± 5.7	108.3 ± 1.2#	119.6 ± 3.3
SDNN (msec)			
9.3 ± 0.8	10.2 ± 1.5	6.7 ± 0.6	8.3 ± 0.6
RMSSD (msec)			
4.6 ± 0.5	5.1 ± 0.6	3.2 ± 0.3	3.6 ± 0.3
pNN_6 (%)			
15.9 ± 2.8	18.2 ± 4.3	7.7 ± 1.7	10.5 ± 2.3
**Dark phase**			
N-N Interval (msec)			
101.7 ± 2.2^	103.2 ± 3.5^	95.6 ± 1.4^	97.1 ± 1.3^
SDNN (msec)			
7.6 ± 0.5	7.5 ± 0.6^	5.5 ± 0.3	5.6 ± 0.7^
RMSSD (msec)			
3.7 ± 0.4	4.1 ± 0.3	2.3 ± 0.1	2.3 ± 0.3^#
pNN_6 (%)			
10.7 ± 2.3	13.2 ± 2.0	3.5 ± 0.8	3.3 ± 1.3

Values are mean ± SEM (ND, *n* = 8; ND+TRF, *n* = 5; HFD, *n* = 8; HFD+TRF, n = 6)
TOD, Time of Day (light or dark phase); TOF, Time of Feeding (at libitum or TRF); SDNN, Standard Deviation of the N-N interval; RMSSD, root mean square of successive differences between normal heartbeats; pNN6, percentage of N-N intervals > 6 msec
^P < 0.05 dark compared to light phase
#P < 0.05 normal compared to high fat diet (within TOD and TOF)

3-way ANOVA results between light and dark phases:
N-N Interval (msec): TOD, P < 0.0001; Diet, P = 0.002; TOF, P = 0.025; TOD×Diet, P = 0.19; TOD×TOF, P = 0.015; Diet×TOF, P = 0.78; TOD×Diet×TOF, P = 0.68
SDNN (msec): TOD, P < 0.0001; Diet, P = 0.003; TOF, P = 0.33; TOD×Diet, P = 0.76; TOD×TOF, P = 0.09; Diet×TOF, P = 0.71; TOD×Diet×TOF, P = 0.73
RMSSD (msec): TOD, P < 0.0001; Diet, P = 0.0001; TOF, P = 0.32; TOD×Diet, P = 0.69; TOD×TOF, P = 0.46; Diet×TOF, P = 0.72; TOD×Diet×TOF, P = 0.64
pNN6 (%): TOD, P = <0.0001; Diet, P = 0.0004; TOF, P = 0.37; TOD×Diet, P = 0.79; TOD×TOF, P = 0.53; Diet×TOF, P = 0.79; TOD×Diet×TOF, P = 0.49

Supplemental Table 3. Diurnal water intake and urinary electrolyte measurements

ND	ND+TRF	HFD	HFD+TRF
**Light phase**			
Water intake (mL/12 hr)			
1.0 ± 0.1	0.7 ± 0.1	0.8 ± 0.1	0.7 ± 0.0
Urine volume (mL/12 hr)			
0.1 ± 0.0	0.2 ± 0.0	0.1 ± 0.0	0.1 ± 0.0
Na^+ excretion (µEq/12hr)			
14.5 ± 5.0	10.6 ± 2.6	10.6 ± 6.1	7.2 ± 2.3
K^+ excretion (µEq/12hr)			
49.0 ± 11.6	39.3 ± 9.8	24.2 ± 8.5	25.9 ± 6.6
**Dark phase**			
Water intake (mL/12 hr)			
1.8 ± 0.1†	2.5 ± 0.2*††	2.0 ± 0.2$^{£}$	2.2 ± 0.2$^{££}$
Urine volume (mL/12 hr)			
0.5 ± 0.1†	0.5 ± 0.1††	0.8 ± 0.1$^{£}$	0.5 ± 0.1$^{££}$
Na^+ excretion (µEq/12hr)			
61.1 ± 22.1	82.5 ± 37.9	69.2 ± 35.4	73.7 ± 34.8
K^+ excretion (µEq/12hr)			
176.6 ± 28.1$^{\#}$	186.6 ± 41.8††	166.6 ± 31.5$^{£}$	189.3 ± 44.4$^{££}$

Values are mean ± SEM; n = 8–11.
*P < 0.05 vs. ND dark phase
†P < 0.05 ND light phase vs. ND dark phase
††P < 0.05 ND+TRF light phase vs. ND+TRF dark phase
£P < 0.05 HFD light phase vs. HFD dark phase
££P < 0.05 HFD+TRF light phase vs. HFD+TRF dark phase
#P = 0.0512 ND light phase vs. ND dark phase

Supplemental Table 4. Cosinor analysis of plasma metabolites

	Rhythmicity			Cosinor Parameters		
Parameter	Diet	R^2	*P* value	Mesor	Amplitude	Phase
Nitrite	ND	0.061	0.430	-	-	-
	ND+TRF	0.136	0.139	-	-	-
	HFD	0.169	0.056	-	-	-
	HFD+TRF	0.186	0.069	-	-	-
Nitrate	ND	0.166	0.072	-	-	-
	ND+TRF	0.386	0.001	19.09	12.21	9.35
	HFD	0.106	0.175	-	-	-
	HFD+TRF	0.322	0.005	17.46	9.36	9.18
IGF-1	ND	0.033	0.625	-	-	-
	ND+TRF	0.357	0.001	246.95	68.90	17.06
	HFD	0.057	0.394	-	-	-
	HFD+TRF	0.238	0.015	306.56[€]	55.17	6.38[€]
β-HB	ND	0.279	0.006	0.16	0.02	16.77
	ND+TRF	0.112	0.170	-	-	-
	HFD	0.195	0.034	0.17	0.02	19.37
	HFD+TRF	0.369	0.001	0.17	0.02	18.88
NEFA	ND	0.203	0.024	287.88	80.72	22.59
	ND+TRF	0.285	0.005	269.72	106.75	5.40[§]
	HFD	0.146	0.080	-	-	-
	HFD+TRF	0.199	0.026	407.54[€]	71.97	23.59[€]

N = 4–6.
No overlap between 95% confidence intervals was detected for the following comparisons between groups displaying significant rhythms by cosinor analysis:
IGF-1, ND+TRF vs. HFD+TRF: mesor, phase
NEFA, ND vs. ND+TRF: phase; ND+TRF vs. HFD+TRF: mesor, phase
[€] indicates statistical significance for differences between ND+TRF vs. HFD+TRF
[§]indicates statistical significance for differences between ND vs. ND+TRF

Supplemental Table 5. Cosinor analysis of plasma BUN and creatinine

	Rhythmicity			Cosinor Parameters		
Parameter	Diet	R^2	*P* value	Mesor	Amplitude	Phase
BUN	ND	0.070	0.351	-	-	-
	ND+TRF	0.164	0.074	-	-	-
	HFD	0.074	0.326	-	-	-
	HFD+TRF	0.086	0.285	-	-	-
Creatinine	ND	0.176	0.060	-	-	-
	ND+TRF	0.042	0.533	-	-	-
	HFD	0.253	0.015	0.047	0.007	3.38
	HFD+TRF	0.153	0.106	-	-	-

Values are mean ± SEM; *n* = 3–6.

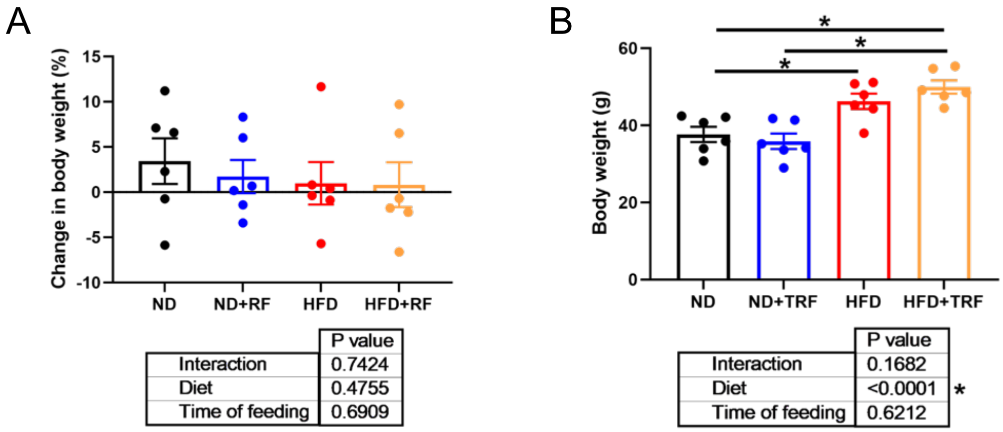

Figure S1. TRF in mice maintained in single housing cages had no significant effect on body weight. To obtain whole body respiration data from individual animals, mice were maintained in single housing units during the final 2 weeks of the study. As in animals maintained in group housing (Figure 1 of manuscript), HFD increased body weight to a similar degree in both ad libitum and TRF groups. (A) change in body weight calculated from immediately before and at the end of the 2-week TRF period. (B) final body weight of animals at the end of the study period. Data were analyzed by two-way ANOVA with individual comparisons being made using Tukey's multiple comparisons tests.

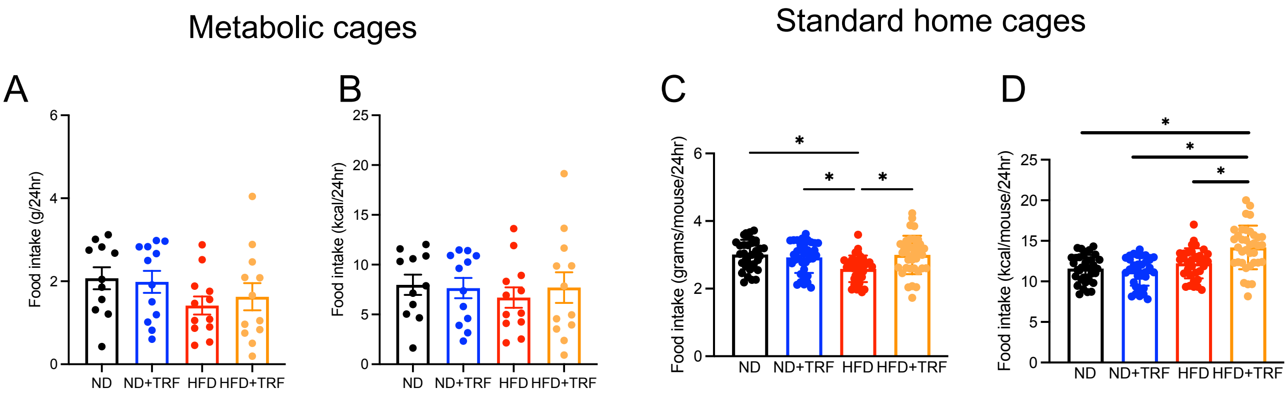

A

	P value
Interaction	0.5842
Time of Feeding	0.8164
Diet	0.0681

B

	P value
Interaction	0.5735
Time of Feeding	0.7763
Diet	0.6039

C

	P value
Interaction	0.0022
Time of Feeding	0.0410
Diet	0.0303

D

	P value
Interaction	0.0013
Time of Feeding	0.0203
Diet	<0.0001

Figure S2. TRF does not reduce 24-hr food intake. Metabolic cages were used to measure 24-hour food intake in grams per day (A) or kcal/day (B) during the final 2 days of the protocol where mice were given normal diet (ND), ND with TRF during the final two weeks, high fat diet (HFD), and HFD with TRF during the final two weeks. Panels C and D show the average daily food intake during the final week of the protocol from all cohorts of animals as measured in home cages. Since mice were group housed, we normalized these data per mouse and per day to aid comparison. Data were analyzed by two-way ANOVA with individual comparisons being made using Tukey's multiple comparisons tests.

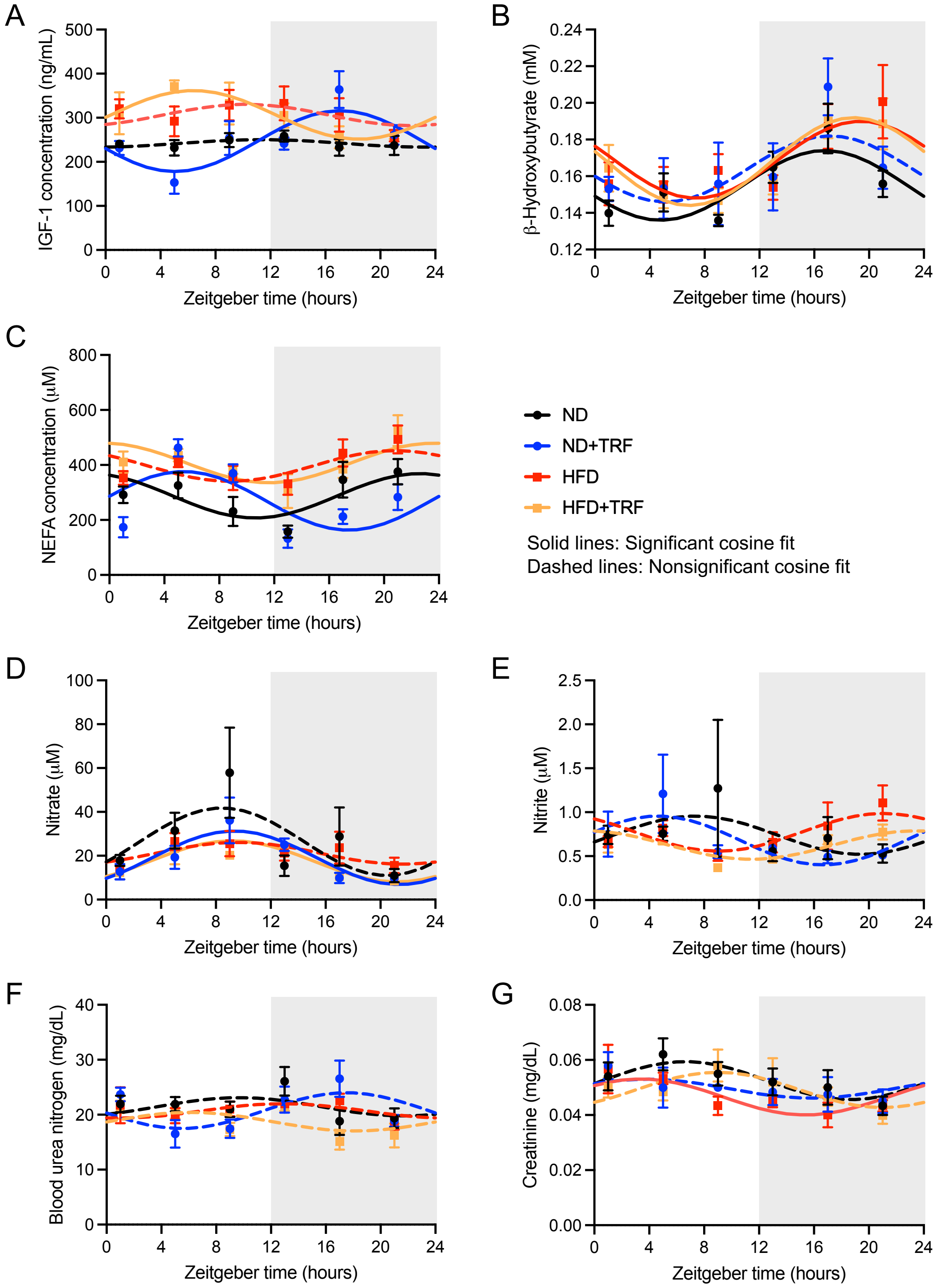

Figure S3. HFD alters rhythms of plasma hormones and metabolites. Blood was collected at 6 time points over a 24-h period from ND and HFD mice fed ad libitum or TRF. Plasma levels of (A) Insulin growth factor-1 (IGF-1), (B) β-hydroxybutyrate (β-HB), (C) Non-esterified free fatty acids (NEFA), (D) Nitrite, (E) Nitrate, (F) Blood urea nitrogen (BUN), and (G) Creatinine were measured at 6 time points. Cosinor analysis was used to determine rhythmicity and circadian parameters (Table S3, S4).

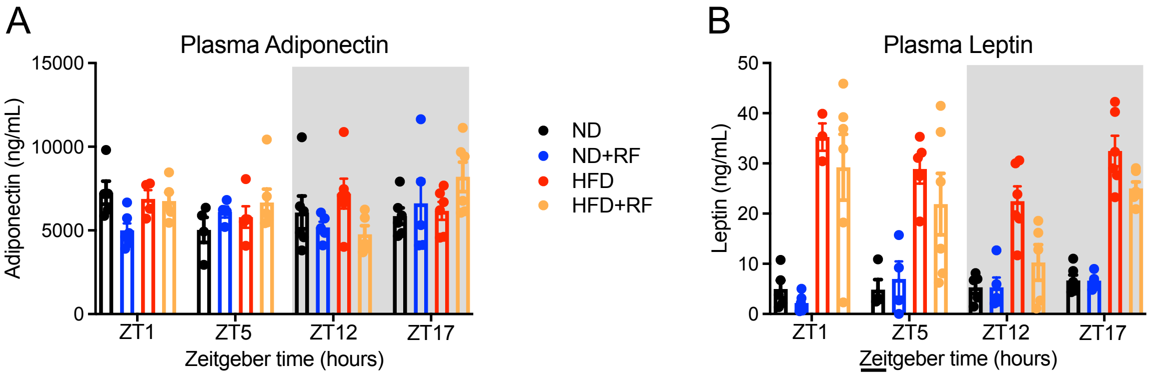

	P value	
Time of day	0.2468	
Diet	0.0831	
Time of feeding	0.7498	
Time of day x Diet	0.9540	
Time of day x Time of feeding	0.0082	*
Diet x Time of feeding	0.5830	
Time of day x Diet x Time of feeding	0.3306	

	P value	
Time of day	0.0135	*
Diet	<0.0001	*
Time of feeding	0.0167	*
Time of day x Diet	0.0060	*
Time of day x Time of feeding	0.8966	
Diet x Time of feeding	0.0228	*
Time of day x Diet x Time of feeding	0.8312	

Figure S4. HFD alters rhythms of plasma adiponectin and leptin. Blood was collected at 6 time points over a 24-h period from ND and HFD mice fed ad libitum or TRF. Plasma levels of (A) adiponectin and (B) leptin are shown at 4 time points, with 2 time points during both light and dark periods. Three-way ANOVA was used to compare diet, time of feeding, and time of day. Symbols indicate statistically significant differences (P<0.05) for post hoc tests between groups at each time point vs. † P<0.05 ND vs. HFD; *P<0.05 HFD vs. HFD+TRF.

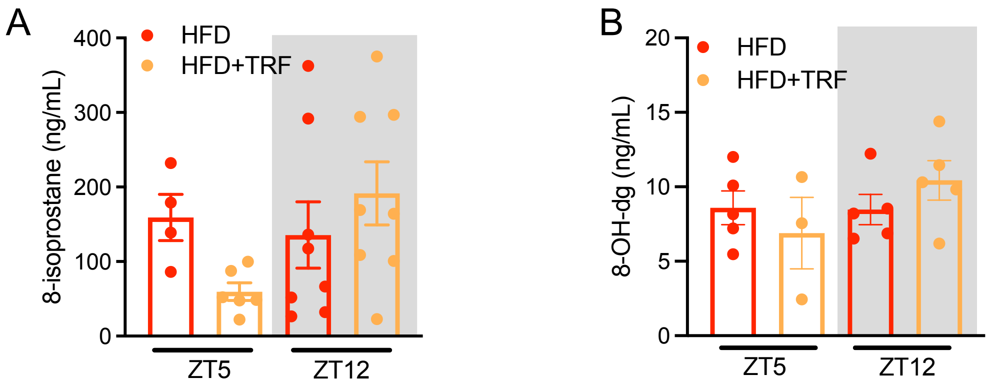

	P value
Interaction	0.0740
Time of day	0.2044
Diet	0.6031

	P value
Interaction	0.2088
Time of day	0.2375
Time of feeding	0.9275

Figure S5. HFD with and without TRF had no significant effects on (A) plasma 8-isoprostane and (B) 8-Hydroxydeoxyguanosine (8-OHdG). Two-way ANOVA was used to compare time of day and time of feeding with Sidak's post hoc test.
